# Attention-driven YOLOv11n–DeiT model for enhanced detection of tomato leaf diseases

**DOI:** 10.1038/s41598-026-64897-8

**Published:** 2026-08-09

**Authors:** Yasmin M. Alsakar, Nehal A. Sakr, Mohammed Elmogy

**Affiliations:** Information Technology Department, Faculty of Computers and Information, Mansoura University, Mansoura, 35516 Egypt

**Keywords:** Tomato leaf disease, YOLO11N, Deit Transformer, Deep learning, Genetics algorithm, Computational biology and bioinformatics, Engineering, Mathematics and computing, Plant sciences

## Abstract

The tomato plant is considered one of the most important crops in the world, yet it is vulnerable to various diseases that affect crop quality and agricultural productivity. These challenges have driven the need for an efficient and intelligent plant disease detection system. With the development of computer vision and artificial intelligence, this proposed methodology based on deep learning for tomato leaf diseases has been presented. Two public datasets: Taiwan DS with nine classes and Tomato Leaf Diseases Detection Computer Vision Dataset (TLDDCV DS) with seven classes have been used to test this system. This system begins with plant image processing, which includes gamma correction and bilateral filtering, to enhance image quality and clarity while preserving key disease features. Then, a genetic metaheuristic algorithm was used to automatically select the most significant hyperparameters, further optimizing both processing time and accuracy. After that, the tomato leaf disease detection applies the You Only Look Once version 11 Nano (YOLOv11n) model. The YOLOv11n backbone is edited through a Data-efficient Image Transformer (DeiT) to improve the system’s capacity for learning global contextual information and long-range dependencies. Experimental results demonstrate that the proposed system outperforms existing methods. It achieved an average mAP@50 of 97.8%, mAP@50-95 of 93.4%, precision of 97.3%, recall of 93.8%, and F1-score of 95.5% on the Taiwan dataset. Additionally, it achieved an average mAP@50 of 87%, mAP@50-95 of 48%, precision of 83.9%, recall of 70.3%, and F1-score of 76.4% on the TLDDCV dataset. These results demonstrate the generalizability and effectiveness of the proposed system in real-world agricultural situations.

## Introduction

Tomato is the most widely consumed and economically significant vegetable crop worldwide, second only to potato in global importance^1,2^. It is used as both a common dietary staple and a fruit-like ingredient, making it highly versatile for various processing and consumption methods. Originating from South America, the Solanum genus has undergone widespread diversification and cultivation^3^. In addition to being a primary lycopene dietary source, an antioxidant associated with a lower risk of cardiovascular illnesses and some types of cancer, tomatoes are also a beneficial source of other essential nutrients, such as vitamin C, folate, potassium, and vitamin K^4^.

Morphologically, the tomato plant is distinguished by compound, serrated leaves and glandular trichomes. With high genetic diversity encompassing thirteen closely related species and several Solanum relatives with varied mating systems, tomatoes are being used as model organisms to research vitamin production, hormone activity, and fruit ripening. Although there is an increase in global tomato cultivation and yields, the industry faces many challenges due to the frequent occurrence of various diseases. These plant diseases represent a significant barrier to achieving high and stable productivity. Therefore, the advancement of precise methods for early detection of tomato leaf diseases is essential to maintain plant health and enhance both quality and yield.

Furthermore, the recurrent occurrence of plant diseases often necessitates extensive pesticide use, which raises environmental concerns and increases production costs^5,6^. To handle these challenges, the advancement of disease-resistant strategies and efficient diagnostic methods is essential. To promote global food security and minimize crop losses, timely and accurate classification of tomato diseases is critical, particularly in the face of a rapidly increasing population^7–9^.

Standard diagnostic techniques, such as expert visual assessments and laboratory-based pathogen detection, are widely used but have significant drawbacks^10,11^. These techniques are time-consuming, labor-intensive, and dependent on personal skills, which limits their scalability and applicability in large-scale agricultural environments^12^. In contrast, more advancements in deep learning (DL) have provided essential alternatives for plant disease detection. DL models such as convolutional neural networks (CNNs) facilitate accurate analysis of complex image data^13,14^. These methods offer several benefits, including automation, adaptability to various environmental conditions, and efficient handling of large-scale datasets. Through enabling real-time diagnosis with minimal human intervention, deep learning holds the potential to advance sustainable agricultural practices and transform crop monitoring.

Identification various tomato leaf diseases through a single image is an essential task in modern agriculture, because tomato plants are commonly affected by many diseases simultaneously, such as: bacterial spot (BS), bacterial blight (BB), late blight (LB), leaf mold (LM), leaf miner (LMI), black spot (BlS), bean rust (BR), bean mosaic virus (BMv), early blight (EB), septoria leaf spot (SLS), spider mites (SM), mosaic virus (MV), target spot (TS), snthracnose (AN) and, yellow leaf curl virus (YLCV). Precise disease detection enables targeted treatment, early intervention, and helps prevent the spread of infections, thereby reducing economic losses and enhancing crop yield. Object detection techniques have proven to be significantly effective in this task, as they can both localize and classify various disease symptoms in the same plant image^15,16^. Table 1 presents the plant disease symptoms and their corresponding characteristics. Alongside YOLO^17–19^, multiple object detection models, such as single shot multiBox detector (SSD)^20^, Faster R-CNN^21^, and RetinaNet^22^, have been used in agricultural disease identification and detection. These methods differ in processing speed and accuracy, providing flexibility for various applications, whether in field identification via drones and mobile devices or high-resolution analysis in controlled environments.

The quick development of artificial intelligence, especially in DL, has transformed the landscape of tomato leaf disease detection. CNN, hybrid architectures, and attention-based models enable scalable and robust solutions that are responsible for handling more complex visual data. These methods can handle various environmental conditions, as leaf occlusion, lighting changes, or overlapping symptoms. Due to the increasing availability of annotated datasets and computational resources, DL-based detection systems are becoming more practical and accessible for real-world use. The AI-driven techniques used in agriculture not only enhance the efficiency and accuracy of disease monitoring but also play a crucial role in supporting global food security and sustainable farming practices^23–25^.

The motivation behind this paper stems from the rapid progress in object detection algorithms, particularly the continuous improvements and newer versions of the YOLO model, which enhance detection efficiency and accuracy. Furthermore, the emergence of transformer-based architectures has a remarkable capability in capturing both global and local features, making them highly effective for identifying tomato leaf diseases. Building on the advancements in these technologies, this paper aims to enhance the accuracy and efficiency of tomato plant disease classification while addressing existing challenges. The fundamental contributions of this paper are summarized as follows:

- Solving the problems of noise in RGB images and uncontrolled illumination through using gamma correction, edge-preserving bilateral filtering, improving image clarity, and emphasizing disease-specific patterns to enhance feature extraction quality.
- Automated hyperparameter optimization using a genetic meta-heuristic algorithm reduces manual tuning time and strengthens model performance.
- Enhancing the detection of fine-grained and subtle disease symptoms through editing the You Only Look Once version 11 Nano (YOLOv11n) architecture with a DeiT transformer-based backbone, which employs global attention to capture higher contextual features.
- Validating the effectiveness and robustness of the proposed system by applying the full methodology on two public tomato disease datasets, where it achieved superior performance over recent approaches.

The paper is organized into the following sections: The section “Related work” presents a review of related work on tomato leaf disease detection using deep learning and object detection approaches. The section “Proposed methodology” introduces the proposed methodology, detailing the YOLO-based framework developed for disease detection. The section “Experimental settings” describes the experimental setup and evaluation process, and provides a comparative analysis of the model’s performance. The section “Experimental results and discussion” discusses the results, analyzing the findings and highlighting the advantages and disadvantages of the proposed approach. Finally, the section Conclusion” concludes the paper with a summary of the key contributions and potential directions for future research. Table 2 provides a detailed list of the abbreviations utilized in this study.

**Table 1:** The plant disease symptoms and characteristics.

Image	Symptom/Characteristic	Image	Symptom/Characteristic
	Marked by concentric ringsand a surrounding yellow zone.		Lesions appear small, dark,and water-soaked in appearance.
	Numerous small, round lightbrown spots with white centers.		Light green or yellowish spotsshow up on the top leaf part.
	Results in tiny, water-soakedlesions on leaves and fruits,often progressing to yellowingand early leaf drop.		No visible lesions, with a greento dark green appearance.
	Plants show symptoms likeyellowing, scorching, leafrolling, and leaf drop.		Mosaic patterns and spotsappear on tomato leaves.
	Leaf edges curl upward(cupping), with thickenedveins, yellowing, and overallleaf distortion.		The leaves display veined,serpentine-like patterns.

**Table 2:** A detailed list of abbreviations utilized in this study.

**Abbreviation**	**Description**	**Abbreviation**	**Description**
An	Anthracnose	AP	Average Precision
BaS	Bacterial Spot	BB	Bacterial Blight
BlS	Black Spot	BMv	Bean Mosaic Virus
BR	Bean Rust	CNN	Convolutional Neural Networks
Deit	Data-efficient Image Transformer	EB	Early Blight
FN	False Negatives	FP	False Positives
HE	Healthy	IOU	Intersection Over Union
LB	Late Blight	LMI	Leaf Miner
LM	Leaf Mold	mAP	Mean Average Precision
ML	Machine Learning	MV	Mosaic Virus
PM	Powdery Mildew (PM)	SBCE	Scaled Binary Cross Entropy
SLS	Septoria Leaf Spot	SM	Spider Mites
SSD	Single Shot MultiBox Detector	SPPF	Spatial Pyramid Pooling Fast
TN	True Negatives	TP	True Positives
TS	Target Spot	VSR	Vector Similarity Regularization
YOLOv11n	You Only Look Once version 11 Nano	YLCV	Yellow Leaf Curl Virus

## Related work

This section discusses several research studies related to tomato leaf diseases, utilizing YOLO and other detection algorithms. For each research, its proposed methodology, key contributions, and the advantages and disadvantages have been summarized. The purpose is to present a comprehensive overview of the existing solutions in this domain and to identify gaps or areas for potential improvement. Table 3 represents a comparison between these studies.

Gomez et al.^26^ utilized an augmented dataset of leaf and pod images from African and Colombian disease hotspots, covering five major diseases: LS, BB, BMV, BR, and AN, with pod images available only for ALS. It applied YOLOv7, YOLOv8, and YOLO-NAS models to multi-level annotations, finding YOLO-NAS superior for leaf detection with a mAP of 97.9% and a recall of 98.8%, while YOLOv7/v8 excelled in pod analysis (> 95% mAP), although micro-annotations underperformed consistently. The optimized YOLO-NAS model was successfully deployed via an Android app, achieving an accuracy of 90% on unseen field data. This demonstrates an efficient DLOps pipeline that enables rapid disease diagnosis and timely farmer interventions to improve bean productivity.

Zhang et al.^27^ introduced an optimized, lightweight object detection model featuring an enhanced backbone architecture, which significantly improves feature extraction for small-scale objects compared to the original YOLOv8 framework. A comprehensive evaluation was conducted comparing the baseline YOLOv8 with the proposed model across multiple input resolutions and architectural scales. Its experiments evaluated on a seven-class tomato plant leaf diseases dataset with TS, BlS, EB, BS, LB, HE, and LM outperformed YOLOv8l by 1.8% in mAP50 while having a % reduction in parameter count.

Kohut et al.^28^ applied DL models for tomato leaf diseases detection, comparing EfficientDetV2, Faster R-CNN, and YOLOv8 variants. YOLOv8n achieved the highest results with a mAP of 96.5%, while maintaining low computational needs, making it well-suited for deployment in mobile or field environments. This research focuses on DL’s capability to enhance sustainable agriculture by real-time detection and presenting future processes using IoT systems and drone technology. This methodology enhanced its adaptability in agricultural environments through dataset expansion and the exploration of alternative models.

Wang et al.^29^ proposed a tomato leaf disease detection system through combining attention mechanisms and multi-scale feature fusion into YOLOv6. This methodology utilized CBAM to enhance lesion feature extraction and limit environmental interference, while the BiRepGFPN module improved small lesion and multi-scale fusion detection. When evaluated on the PlantDoc dataset, it outperformed many YOLO variants (YOLOX to YOLOv8), achieving mAP advancements of 2.7–11.8%. In addition, when evaluated on the tomato disease dataset, it achieved mAP of 93.8%, precision of 92.9%, and recall of 95.2%, representing 2.3–4.0% gains over the baseline.

Bachri et al.^30^ used the Faster R-CNN model to detect eight tomato diseases, including LB, EB, LM, MV, LMI, LS, SM, YLCV, and HE. It was evaluated using a dataset collected from Kaggle, Google Images, and the Roboflow Universe. This methodology applied processing techniques (tiling, collage, resizing, cropping) and data augmentation (rotation, flipping, exposure/hue adjustments). The X101-FPN base algorithm achieved an 87.01% MAP in validation, demonstrating the highest disease detection capabilities. This methodology yielded better results in identifying agricultural defects while maintaining generalization performance.

Matsui et al.^31^ enhanced loss functions in the YOLO model for plant leaf diseases detection in agricultural datasets. This research relied on two loss functions: scaled binary cross entropy (SBCE) for small-object detection and vector similarity regularization (VSR) for ripeness classification. It achieved the highest results, with a mAP of 70.21% (up from 61.81%), an F1 score of 71% (from 61%), and a mIoU of 66.44% (from 65.03%), through the application of the YOLOv7-tiny model. These developments demonstrated the potential of AI to streamline agricultural efficiency.

Lee et al.^32^ utilized YOLOv11 for detecting tomato leaf diseases. This methodology was evaluated using an improved dataset that included HE, LB, EB, YLCV, SLS, LM, MV, SM, TS, and BS. After fine-tuning the variants, the YOLOv11m model showed superior baseline performance, with a fitness of 0.98885, recall of 0.98597, precision of 0.99104, and mAP@0.5 of 0.99197. Sequential optimization using OFAT through seven key parameters, combined with a 100-configuration random search, yielded the C47 model, which demonstrated consistent performance gains – a fitness increase of +0.39%, precision of +0.09%, recall of +0.76%, and mAP@.5 of +0.07%.

Joshi et al.^33^ proposed a detection system for tomato leaf diseases through developing an automatic system based on the YOLOv8n model. Although tomato plants were highly vulnerable to multiple diseases, traditional detection methods have shown limited efficiency in large-scale farming. The authors handled data security through hybrid augmentation, expanding their PlantDoc dataset from 737 to 6,696 images. Their methodology achieved a precision of 97% and an mAP of 96.5% in detecting seven disease classes, outperforming related approaches on the PlantVillage dataset. This flexible methodology showed potential for broader agricultural applications.

Abulizi et al.^34^ used a DM-YOLO detection model based on YOLOv9. It was evaluated on a dataset with nine classes: LB, LM, SM, MV, SLS, YLCV, EB, HE, and LMI. This methodology consisted of two key enhancements: (1) DySample for better background interference suppression and small lesion feature extraction, and (2) MPDIoU loss for enhanced overlapping lesion margin localization. This proposed methodology yielded experimental results with an average precision (AP) of 95.1%, precision (P) of 92.5%, and mean average precision (mAP) of 86.4%, outperforming the baseline YOLOv9 by 1.7%, 3%, and 1.4%, respectively. These experimental results highlight the robust detection performance of DM-YOLO and its potential to enhance disease management applications and smart agriculture.

AlHusaini et al.^35^ proposed a tomato leaf disease detection system using YOLOv11 on a dataset of 737 leaf images. The improved model architecture combined C2 modules for extracting features, SPPF layers for multi-scale detection, and optimized NMS techniques. This methodology achieved strong performance metrics, with a precision of 80%, an accuracy of 75.6%, a recall of 77%, and a mAP@50 of 75.6%. This methodology demonstrated strong performance in identifying tomato leaf disease, maintaining accuracy even under challenging conditions involving environmental variations and overlapping objects. These experimental results highlight YOLOv11’s ability as a scalable, practical solution for precision agriculture, offering reliable disease monitoring while maintaining operational efficiency. Liu et al.^16^ proposed a method for detecting tomato leaf diseases. It applied more than one models, one of them was swin transformer, an improved transformer-based model, used in multiple computer vision tasks. This paper used Swin in the neck layer instead of HCMamba module because swin has strong feature extraction and representation capabilities which was suitable for complex image data and for capturing long-range dependencies. This method achieved 0.839 and 0.676 for mAP50 and mAP50-95 respectively.

Although the effectiveness of related research in using YOLO-based and other DL models for tomato leaf disease identification, various limitations remain unaddressed. (1) Multiple YOLO-based models depend on standard backbones without modification, which limits their capability to extract more significant features, particularly in fine-grained disease identification. (2) Most recent models do not incorporate preprocessing techniques, making them less robust when dealing with low-contrast, noisy, or poorly illuminated images. (3) Selecting parameters in prior research is often heuristic-based or manual, which has a negative effect on the system’s generalization and performance. (4) Fewer researchers concentrated on the integration of transformer architectures with the YOLO model to improve global context understanding and feature representation. Therefore, there is a need for an adaptive and enhanced object detection framework that can address these limitations.

To address previous limitations, this proposed methodology presents a new system for identifying tomato leaf diseases that enhances both efficiency and accuracy. In contrast to previous papers that relied on standard YOLO models without modification, this proposed methodology presents an enhanced YOLOv11n architecture, incorporating a DeiT transformer backbone to improve feature representation. This system consists of basic steps such as: (1) image preprocessing by applying gamma correction and bilateral filtering to highlight disease regions and enhance image quality. (2) Optimizing parameters based on a meta-heuristic algorithm to fine-tune model settings automatically. (3) Detecting tomato diseases using the edited YOLOv11n model. (4) Evaluating performance and making comparisons with previous methods on two public datasets. A thorough explanation of each step in the suggested system is provided in the following section.

**Table 3:** An overview of previous studies on the use of several methods to identify tomato plant leaf diseases.

Publication	Detection Methodology	Detected Diseases	Dataset	mAP50%	mAP50-95%	Strengths	Limitations
Gomez et al. ^26^ (2024)	YOLO-NAS	LS, BB, BMV, BR, and AN	Private	97.9	—	High mAP50	Low quality images
Zhang et al. ^27^ (2024)	YOLOv8m with EfficientNetB0	BS, EB, HE, LB, LM, TS, and BS	Tomato DS^36^	79	52.9	Reduced parameters by 74%	Low-resolution images
Kohut et al. ^28^ (2024)	Faster RCNN	EB, LB, LMI, LM, MV, LS, SM, YLCV, and HE	Tomato DS^37^	96.5	84	High accuracy	Low quality images
Wang et al. ^29^ (2024)	YOLOv6	EB, LB, LMI, LM, MV, LS, SM, YLCV, and HE	Tomato DS^37^	93.8	—	Strong generalization	High computational cost
Bachri et al. ^30^ (2024)	Faster RCNN	EB, LB, LMI, LM, MV, LS, SM, YLCV, and HE	Tomato DS^37^	87.01	—	Fast methodology	Low quality images
Matsui et al. ^31^ (2025)	YOLOv7-tiny	BS, EB, HE, LB, LM, TS, and BS	Tomato DS^36^	70.2	—	Robustness to Small Objects	Low accuracy
Lee et al. ^32^ (2025)	YOLOv11m	LB, HE, EB, SLS, YLCV, BS, TS, MV, and LM, SM	Tomato DS^38^	99.104	98.59	High Accuracy	High computational cost
Joshi et al. ^33^ (2025)	YOLOv8n	BS, EB, HE, LB, LM, TS, and BS	Tomato DS^36^	96.5	—	High accuracy	High computational cost
Abulizi et al. ^34^ (2025)	Improved YOLO9	EB, LB, LMI, LM, MV, LS, SM, YLCV, and HE	Tomato DS^37^	95.1	86.4	Improved Feature Extraction	High computational cost
AlHusaini et al. ^35^ (2025)	YOLOv11	BS, EB, HE, LB, LM, TS, and BS	Tomato DS^36^	75.6	—	All critical metrics included	Low accuracy
Liu et al. ^16^ (2025)	Swin	EB, LB, LMI, LM, MV, LS, SM, YLCV, and HE	Tomato DS^37^	84.6	69.3	Using transformer to extract local features	Low accuracy

## Proposed methodology

**Fig. 1:**
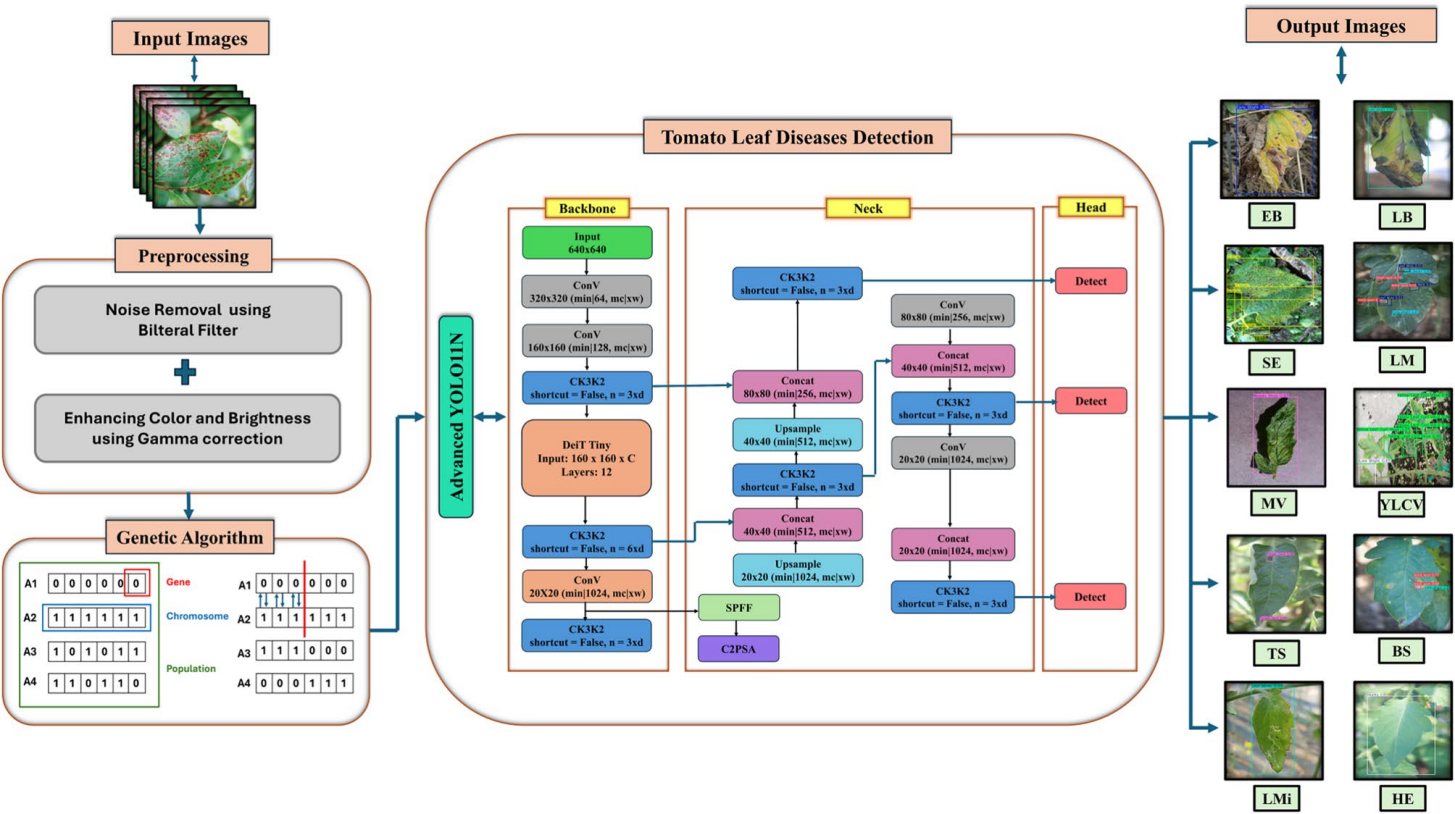
The framework for tomato leaf disease detection using enhanced YOLO11N architecture.

This section discusses the proposed methodology, which has been designed to create a robust and effective system for detecting and identifying tomato leaf diseases. This methodology comprises various key components. Firstly, the input image preparation involves two publicly available datasets: Taiwan DS (9 classes) and Tomato Leaf Diseases Detection Computer Vision Dataset (TLDDCV DS) (7 classes), ensuring that the proposed methodology has been evaluated across various class distributions and conditions. Next, image processing has been applied, which includes gamma correction to highlight significant disease features and improve image contrast, as well as bilateral filtering to remove noise while preserving edges. Then, a genetic metaheuristic optimization algorithm was applied to automatically select the best model parameters without manual tuning. Finally, the previous steps have been integrated to apply an improved YOLOv11n algorithm, where its backbone has been modified by incorporating a DeiT transformer. This modification proposes to highlight feature extraction through capturing global context for achieving the highest accuracy in disease detection. Figure 1 shows the framework for tomato leaf disease detection and identification.

### Input images

This section discusses the datasets used in this proposed methodology. Two public datasets for detecting and identifying tomato leaf diseases have been used to evaluate the generalization ability and performance of the proposed methodology. The first dataset consists of nine classes with more complex visual variations and a variety of disease conditions^37^. The second dataset combines seven classes^36^, with many disease types and healthy leaves. These evaluated datasets have been chosen to provide multiple challenges in identification and to ensure the model’s accuracy. Further details about both datasets have been discussed in the experimental setup section.

### Image preprocessing

Image preprocessing is a crucial step in computer vision, applied to enhance image quality and accentuate relevant features for subsequent interpretation and analysis. Effective preprocessing can significantly impact the performance of downstream tasks by reducing noise and improving contrast^39,40^.

In this work, we applied a three-stage preprocessing pipeline consisting of normalization, bilateral filtering, and gamma correction.

1. *Normalization:* It scales the pixel values to a consistent range, converting them to [0, 1]. This stabilizes and accelerates the training of deep learning models, ensures that all input features contribute proportionally, and improves convergence and overall performance:
  1 $$\begin{aligned} I_{norm} = I/255 \end{aligned}$$
2. *Bilateral Filtering:* It effectively reduces noise while preserving edges by combining spatial and intensity domain information^41,42^. This nonlinear filtering approach calculates weighted averages where pixels are weighted based on both their geometric closeness and photometric similarity. Unlike traditional filters like gaussian filtering, it depends on both intensity and spatial proximity differences between pixels. This makes it to smooth homogeneous regions (e.g., healthy leaf areas) while preserving sharp edges (e.g., disease boundaries). Finally, it improves the lesion regions visibility and prevent the critical features loss that are very important for accurate diseases classification. Mathematically, for an image *I*, the bilateral filtered output $I_{BF}$ at pixel *p* is given by:
  2 $$\begin{aligned} F(p) = \frac{1}{Z_p} \sum _{r \in \Theta } K_{\alpha }(\Vert p-r\Vert ) \, K_{\beta }(|J(p)-J(r)|) \, J(r) \end{aligned}$$
  where $K_{\alpha }$ and $K_{\beta }$ are spatial and range Gaussian kernels, *Θ* is the neighborhood window, and $Z_p$ is the normalization factor. We applied edge-preserving smoothing using spatial ($\sigma _s=3$) and range ($\sigma _r=0.1$) parameters with a *5× 5* window.
3. *Gamma Correction:* It is used to optimize brightness and contrast. Gamma correction is a nonlinear operation that edits the values of luminance according to a power-law relationship^43,44^. The transformation is especially designed to improve details in both overexposed and shadowed regions. It improves low-intensity regions and standarizes illumination changes caused by inconsistent lighting conditions during image acquisition. This is very useful in plant leaf images, where diseases symptoms may appear in darker or less visible regions. Through enhancing the contrast between healthy and diseased areas, gamma correction algorithm makes better feature extraction and enhances the discriminative model capability. The operation has been defined as:
  3 $$\begin{aligned} I_{\gamma }(x,y) = I_{BF}(x,y)^\gamma \end{aligned}$$
  where *γ> 1* decreases the image brightness to highlight details, while *γ < 1* increases the image brightness to enhance shadow visibility. The contrast has been improved by *γ =1.3* to reveal image details:
  4 $$\begin{aligned} I_{out} = I_{BF}^{1.3} \end{aligned}$$

**Fig. 2:**
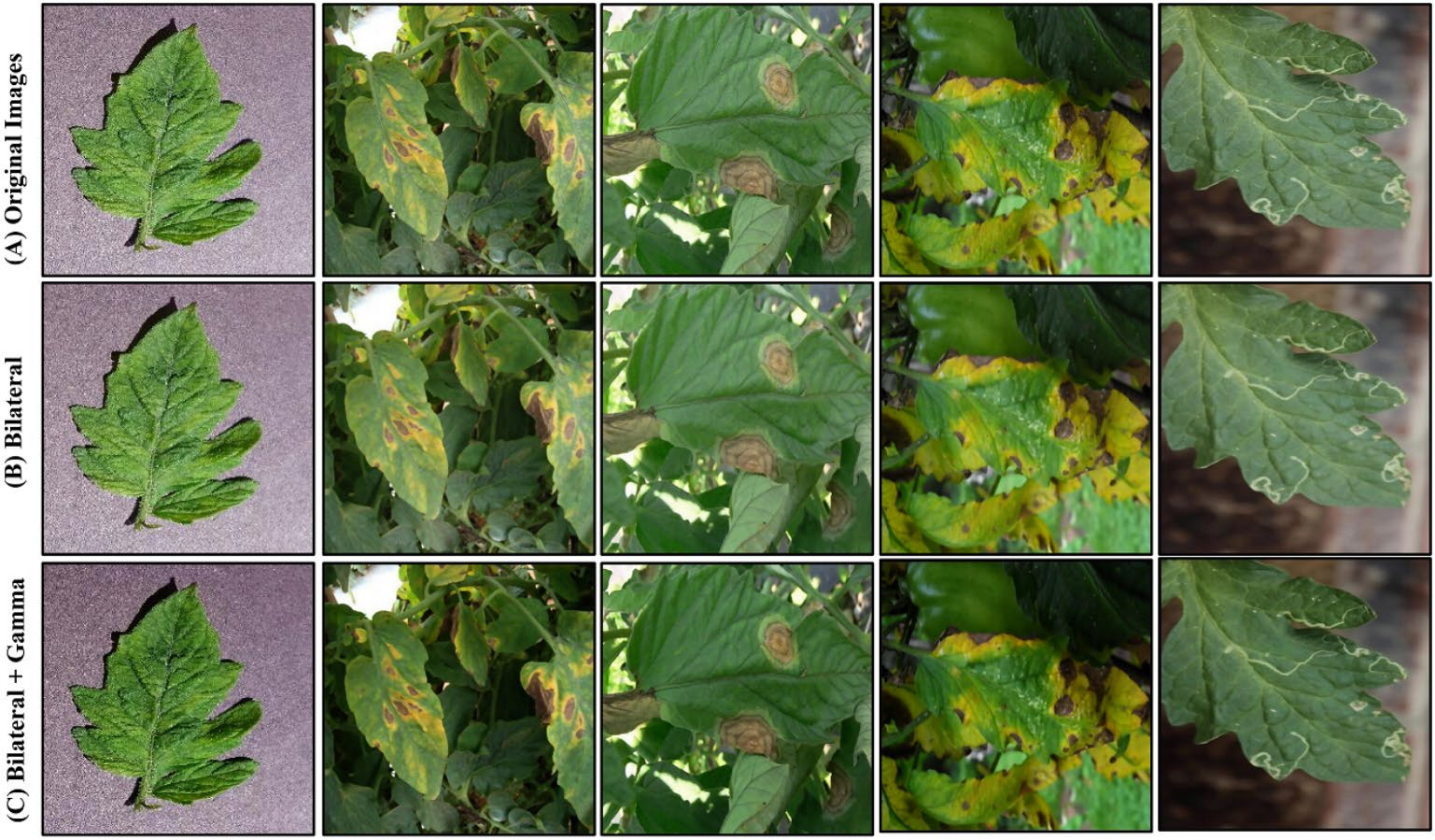
Applying Bilateral and gamma correction on tomato image samples from Taiwan and TLDDCV datasets.

Figure 2 illustrates the progressive improvement across the processing stages, demonstrating enhanced visual quality while preserving essential image features.

### Automated hyperparameters selection

Choosing the primary hyperparameters plays a crucial role in enhancing the performance of ML and DL models. In this work, the genetic algorithm (GA), a metaheuristic technique modeled after the natural selection process^45–48^, is utilized to optimize the hyperparameters of the YOLO11n model for tomato leaf diseases. The performance of detection models, such as YOLO11n, is highly dependent on several hyperparameters. Instead of manually tuning them, the GA was used to automatically explore the optimal configuration.

The algorithm has been used to optimize primary model hyperparameters, including the initial learning rate (lr0), learning rate factor (lrf), momentum (momentum), weight decay (weight_decay), intersection-over-union threshold (iou), warmup epochs (warmup_epochs), bounding box loss gain (box), and classification loss gain (cls). Each individual (chromosome) in the population is a candidate solution that is encoded as a vector, as defined in Eq. (5).

5 $$\begin{aligned} \textbf{x}_i = [\text {lr0}_i, \text {lrf}_i, \text {momentum}_i, \text {weight}\_\text {decay}_i, \text {iou}_i, \text {warmup}\_\text {epochs}_i, \text {box}_i, \text {cls}_i] \end{aligned}$$

Each solution’s fitness was evaluated using the mean Average Precision (mAP) achieved by the YOLOv1N model on the validation set:

6 $$\begin{aligned} f(\textbf{x}_i) = \text {mAP}(\textbf{x}_i) \end{aligned}$$

The GA process includes selection based on fitness:

7 $$\begin{aligned} P(\textbf{x}_i) = \frac{f(\textbf{x}_i)}{\sum _{j=1}^{N} f(\textbf{x}_j)} \end{aligned}$$

Some of the benefits that the Genetic Algorithm brings to hyperparameter optimization for models such as YOLO11n include the following. First, the algorithm performs global optimization, meaning that the probability of getting stuck at a local optimum is minimized compared to other approaches such as gradient descent or exhaustive search. Secondly, the Genetic Algorithm is efficient when dealing with high dimensional search space problems, since the algorithm evaluates only a few candidate solutions per generation instead of all potential hyperparameter combinations. Furthermore, the selection, crossover, and mutation operations of the algorithm ensure the quality improvement of candidate solutions during the optimization process. Moreover, the genetic algorithm is highly adaptable to continuous, discrete, and non-linear hyperparameter search spaces. The latter is especially significant for deep learning algorithms since hyperparameters tend to exhibit a complicated non-linear interaction pattern in such cases. Table 4 explains the optimized chosen Hyperparameters using genetic algorithm.

**Table 4:** Optimized Hyperparameters for YOLO11n using Genetic Algorithm.

**Hyperparameter**	**Description**	**Optimal Value**
lr0	Initial learning rate for model training	0.00639
lrf	Final learning rate factor	0.02225
momentum	Momentum for optimizer (helps accelerate gradients in correct direction)	0.8775
weight_decay	Regularization term to avoid overfitting	2.31 *×* 10$^{-5}$
iou	Intersection-over-union threshold for positive predictions	0.6473
warmup_epochs	Warmup epochs number before full learning rate	3.38
box	Bounding box loss gain (weight for box regression loss)	0.1838
cls	Classification loss gain (weight for class prediction loss)	0.3609

In addition, crossover and mutation operations generate new populations iteratively. Through this optimization process, the GA guided the search towards a set of hyperparameters that significantly enhanced the model’s ability to detect tomato leaf diseases in images.

### Object detection using the enhanced YOLO11n model

Ultralytics released YOLOv11 in September 2024, representing a significant development in the YOLO versions^49^. According to its competitor’s advantages, YOLO11 presents various improvements designed to strengthen both performances. It incorporates an improved backbone and neck architecture that greatly enhances feature extraction and object detection accuracy, especially in complex environments.

YOLO11 is a real-time object detection model that follows the single-stage architecture advantages of the YOLO family. There are five variants, namely n, s, m, l, and x, which contain the same overall architecture but differ in network depth and width. Among these, YOLO11n achieves the highest inference speed at the cost of reduced accuracy, whereas YOLO11x provides superior accuracy with slower processing. YOLO11s serves as a middle ground, offering a balanced compromise between detection speed and performance.

In this work, the YOLO11n version has been selected due to its lightweight architecture and high inference speed, making it well-suited for real-time deployment. To further improve detection accuracy and strengthen feature extraction, the original convolution-based backbone of YOLO11n was replaced with the DeiT model. DeiT, a transformer-driven architecture, is recognized for its strong representation learning capabilities, even when trained on relatively small datasets. Incorporating DeiT as the backbone introduces a global attention mechanism, enabling the network to better capture long-range dependencies and contextual information within the input images. The enhanced YOLO11n architecture with the modified backbone is indicated in Fig. 4.

Despite YOLOv11 effectively uses convolutional operations to extract multiscale local features, convolution-based backbones are still limited in global contextual relationships and long-range dependencies within complex images. Therefore, DeiT transformer has been applied which uses a self-attention mechanism that enables the network to capture both local and global representations simultaneously, allowing better understanding of spatial relationships between distant regions in the image. This capability is very especially significant in challenging detection scenarios where disease symptoms may appear with varying scales, irregular shapes, or subtle visual patterns. Therefore, integrating DeiT into the YOLO11n backbone improves the discriminative feature representation and enhances the model’s capability to concentrate on informative regions, leading to more robust detection performance compared with the original convolution-based YOLO11n backbone.

#### Enhanced YOLO11N backbone with data-efficient image transformer (DeiT)

**Fig. 3:**
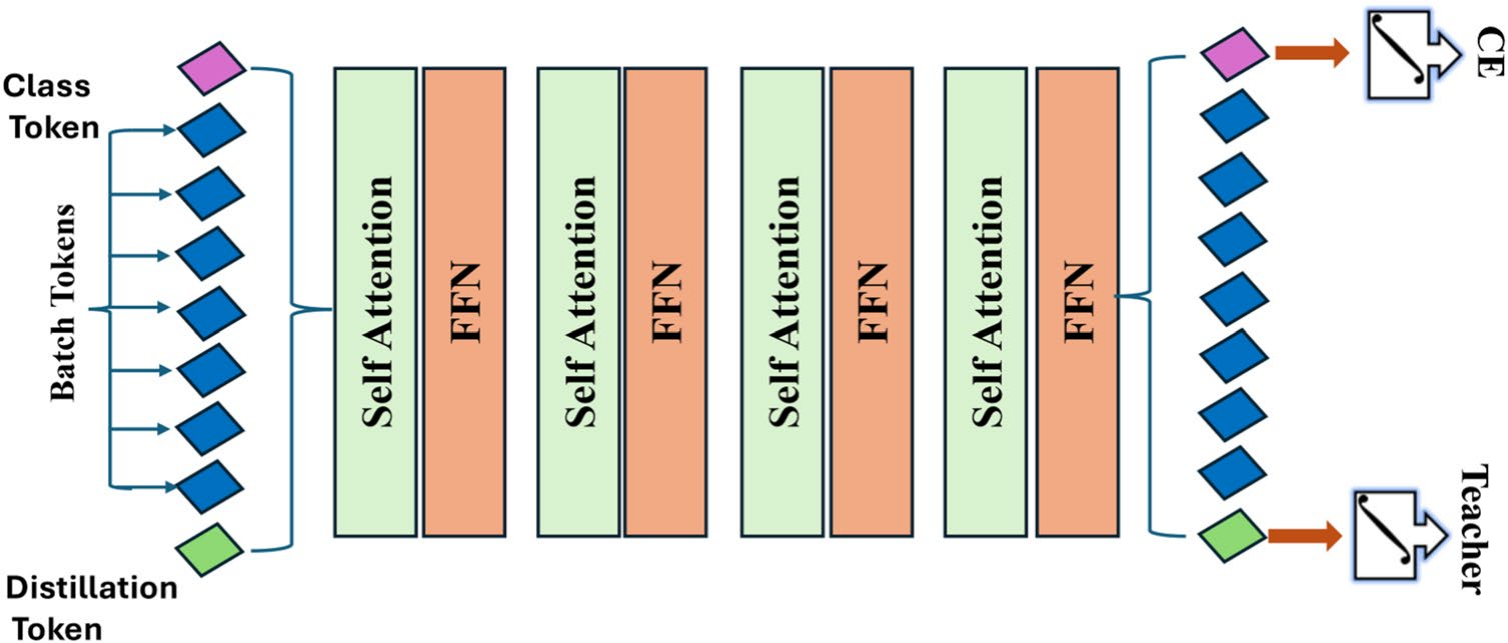
The Deit Transformer architecture.

The DeiT is an enhanced version of the vision transformer (ViT) designed to improve data efficiency by employing a knowledge distillation strategy between a teacher and a student network^50^. Unlike traditional convolutional networks, DeiT treats images as sequences by dividing them into patches that are transformed into tokens. These tokens are then projected into an embedding space and processed through multiple self-attention layers to learn spatial and contextual dependencies. The architecture of DeiT is illustrated in Fig. 3.

We denote the input image as $\textbf{x} \in \mathbb {R}^{H \times W \times C}$, where *H*, *W*, and *C* correspond to the image height, width, and number of channels, correspondingly. The DeiT processing pipeline can be summarized as follows:

- **Patch embedding:** The image is partitioned into non-overlapping patches of size *p × p*, yielding a total number of regions:
  8 $$\begin{aligned} N = \frac{H \times W}{p^2}. \end{aligned}$$
- **Linear embedding:** Every patch is flattened and projected linearly into a feature vector of dimension *d*. The resulting embedded input sequence is:
  9 $$\begin{aligned} \textbf{z}_0 = [\textbf{x}_{cls}; E\textbf{x}_1; E\textbf{x}_2; \ldots ; E\textbf{x}_N] + E_{pos}, \end{aligned}$$
  where $\textbf{x}_{cls}$ is a learnable classification token, *E* is the patch embedding matrix, and $E_{pos}$ denotes the positional embeddings.
- **Self-attention mechanism:** The transformer core is on the multi-head self-attention mechanism, which is computed as:
  10 $$\begin{aligned} \text {Attention}(\textbf{Q}, \textbf{K}, \textbf{V}) = \text {Softmax}\left( \frac{\textbf{Q}\textbf{K}^{\top }}{\sqrt{d_k}}\right) \textbf{V}, \end{aligned}$$
  Here, $\textbf{Q}$, $\textbf{K}$, and $\textbf{V}$ represent the query, key, and value matrices, respectively, while $d_k$ is the key vectors’ dimensionality.

Through feed-forward processes and repeated layers, DeiT produces a rich feature representation $z_{out}$ that captures the input image semantic content^51^. Figure 3 indicates the Deit architecture.

#### YOLO11N neck layer

The YOLOv11n architecture Neck represents an essential role in combining multi-scale backbone features and making them for precise object detection at various resolutions. The Neck uses a combination of upsampling, concatenation, and specialized convolutional blocks to mix feature maps from different levels of the network. One of the key components in this stage is the spatial pyramid pooling - fast (SPPF) module, which combines features across various receptive fields, thereby enhancing the network’s ability to identify objects at multiple scales. This is followed by a C2PSA module, which is used for containing semantic and spatial information while enhancing feature representation.

The upsample operations have been utilized to make spatial resolutions, enabling the concatenation of higher-resolution features from earlier stages. These combined parts then pass through blocks (which contain residual features and shortcut connections) to create a representation while maintaining gradient flow and avoiding vanishing gradients. By frequently creating upsampling and concatenation, the Neck makes a rich and multi-resolution feature set that strengthens the model’s performance on small and medium-sized object detection.

#### YOLO11N head layer

The Head of YOLOv11n architecture is responsible for producing the final object detection outputs, which contain objectness scores, bounding box coordinates, and class probabilities. It consists of three sets of feature maps from the Neck, corresponding to various scales (20*×*20, 40*×*40, 80*×*80), enabling it to identify objects of various sizes.

**Fig. 4:**
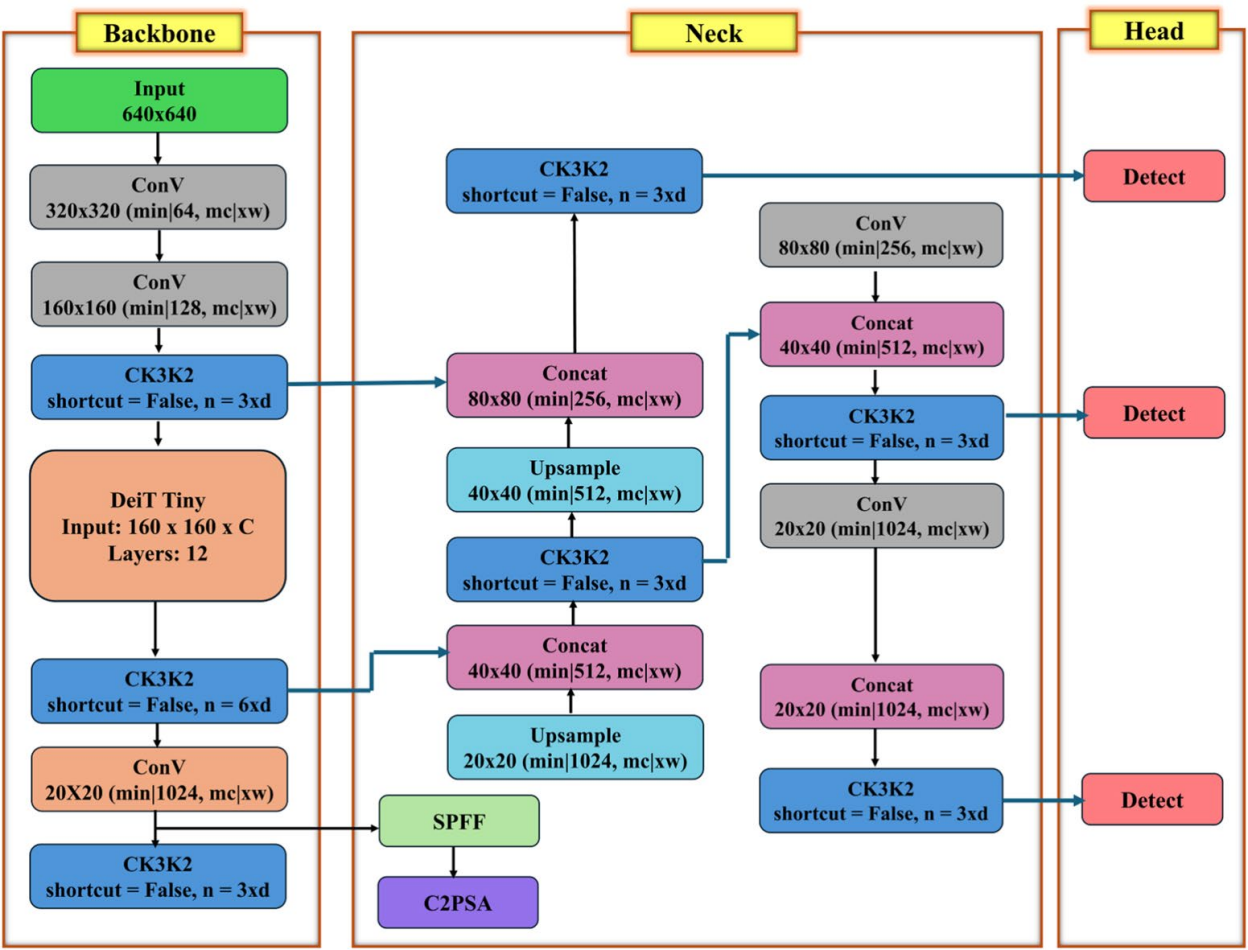
The YOLO11N architecture with enhancement in its backbone with DeiT transformer.

Each of these feature maps has been delivered directly into a Detect layer, which makes predictions using anchor-based or anchor-free mechanisms based on a precise configuration. The detection layers perform 1*×*1 convolutions to generate the specific number of outputs per grid cell, such as bounding box regression values (x, y, w, h), class scores, and objectness confidence. This multi-scale detection stage ensures high performance and accuracy for small, medium, and large objects. Therefore, the parallel multi-head setup makes simultaneous detection at various spatial resolutions, making the YOLOv11n model more efficient in real-time environments. Figure 4 illustrates the YOLO11N architecture with enhancements in its backbone based on the DeiT transformer.

The algorithm 1 presents a comprehensive set of DL steps for automatic detection and identification of tomato diseases. It starts with image processing, where gamma correction and bilateral filter have been applied to improve image quality and contain important disease features. These process images have been resized to fit the YOLOv11n input dimensions. Then, the YOLOv11n backbone has been improved with a DeiT transformer to combine subtle patterns and global contextual information. For hyperparameter optimization, a genetic algorithm has been applied to automatically choose the most effective hyperparameters and reduce manual tuning. The improved model is then trained on two public datasets, namely Taiwan DS and TLDDCV DS, and has been evaluated through multiple metrics, such as mAP@50, mAP@50–95, precision, recall, and F1-score. Finally, the trained model is applied to identify new tomato leaf images into their respective disease classes, demonstrating strong generalization and robustness across varying conditions.

**Algorithm 1:**
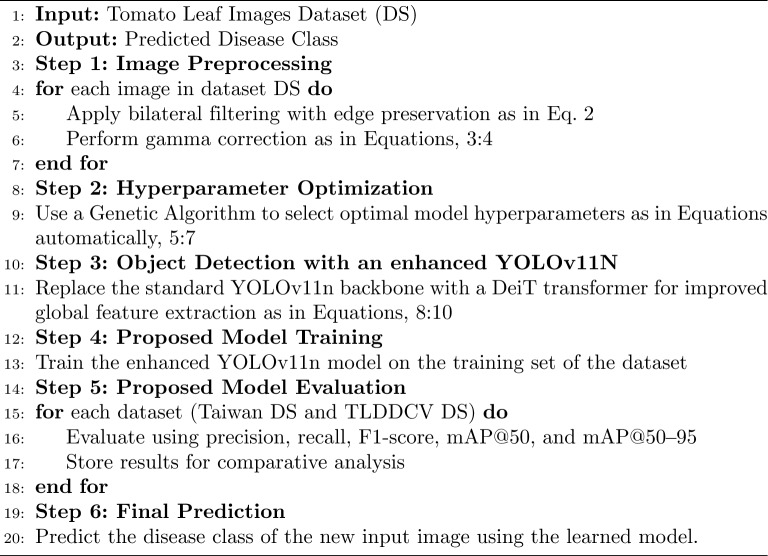
The tomato leaf diseases classification

The model training process was performed using the Ultralytics YOLO framework with clearly specified hyperparameters for reproducibility and reliable testing of the results. The model was trained over 100 epochs with a batch size of 16 and a resolution of 640*×*640 pixels per input image. The AdamW optimizer was utilized as the default optimization technique for efficient training of the deep learning model. The learning rate scheduling was defined by the YOLO training process. Early stopping criteria were initially tested, but based on empirical findings, the convergence of the model was reliably achieved at about 100 epochs with no noticeable improvement of the validation mean Average Precision (mAP). Thus, training was done for 100 epochs with the selection of the best model checkpoint according to validation mAP.

## Experimental settings

The effectiveness of the proposed framework for tomato leaf disease detection has been evaluated using Python, a high-level programming language widely adopted in scientific computing due to its readability, extensive library support, and cross-domain applicability. Python is particularly well-suited for machine learning (ML), computer vision, bioinformatics, and data analytics, making it an ideal choice for this research. To continue model development, model training, and evaluation, many packages have been used, such as NumPy for numerical computations, Pandas for data manipulation, Seaborn and Matplotlib for data visualization, OpenCV for computer vision, cikit-learn for traditional ML models, TensorFlow and Keras for DL models, and PyTorch for additional flexibility in neural network experimentation. Moreover, libraries such as Pillow for image handling and SciPy for advanced scientific computations have been utilized to enhance preprocessing and analysis.

The experimental evaluation was conducted on a system equipped with an Intel(R) Core(TM) i7-9750H CPU @ 2.60 GHz (Lenovo, Beijing, China), 16 GB RAM, and the Microsoft Windows 10 operating system (Microsoft, Redmond, WA, USA). The environment uses Python 3.8+ with Jupyter Notebook for interactive prototyping and testing, ensuring effective debugging and real-time analysis. The next subsection presents a detailed discussion of evaluation metrics, including precision, recall, F1-score, mAP50, and mAP50-95, to comprehensively assess the model’s performance in detecting tomato diseases.

### Performance evaluation metrics

To evaluate the effectiveness of the proposed methodology, Various performance metrics have been utilized. This subsection presents a detailed discussion of the mathematical equations underlying these metrics, including precision, recall, F1-score, mAP50, and mAP50-95. They have been computed based on true positives (TP), which represent the number of instances correctly identified as belonging to a given class, false positives (FP) are the instances incorrectly assigned to a class, and true negatives (TN) are the instances correctly predicted as not belonging to a specific class. Finally, false negatives (FN) represent the number of instances that are incorrectly predicted to be in a class despite actually belonging to it.

- **Precision :** It is the percentage of correctly recognized diseased tomato leaves (TP) to the total tomato leaf images predicted as diseased (FP + TP)^52^. It is calculated by Eq. (11).
  11 $$\begin{aligned} Precision = \frac{TP}{TP+FP} \end{aligned}$$
- **Sensitivity (Recall):** It represents the percentage of correctly recognized diseased tomato leaf classes (TP) to the total number of actual diseased leaf classes, ((TP) + (FN))^53^. It is calculated by Eq. (12).
  12 $$\begin{aligned} Recall = \frac{TP}{FN+TP} \end{aligned}$$
- **Dice Similarity Coeffient (F1-Score):** It is used to assess the system quality^54^, reflecting the trade-off between precision and recall. This metric is particularly useful in cases of class imbalance. It is calculated by Eq. (13).
  13 $$\begin{aligned} F1-Score = \frac{2*TP}{2*TP+FN+FP} \end{aligned}$$
- **Mean Average Precision (mAP): ** It is the mean value of the Average Precision (AP) scores through various disease classes, which represents the overall classification ability of the tested model on the dataset^55^. A higher mAP means that the model’s detection performs better across all disease classes. Eq. (14) is the computation of mAP.
  14 $$\begin{aligned} \text {mAP} = \left( \frac{1}{\text {classes}} \sum _{i=1}^{\text {classes}} \text {AP}_i \right) \times 100\% \end{aligned}$$
  Here, *classes* refers to the number of disease categories. MAP at IoU = 0.50 (mAP50) computes the AP through all classes using a 0.50 intersection-over-union (IoU) threshold. This metric represents the model’s performance in detecting multiple objects when there is a moderate degree of overlap. To have a better comprehensive evaluation, the mean AP over IOU thresholds ranging from 0.50 to 0.95 (mAP50–95) is considered. This metric computes the average precision across multiple IoU thresholds, increasing in steps of 0.05, thereby providing a more thorough assessment of the model’s accuracy in predicting bounding boxes at different overlap levels.
- To evaluate performance through many classes and highlight misclassifications, confusion matrices were applied for every model. The model’s predictions and the true labels are compared in detail using a confusion matrix, which displays the values of TP, FP, FN, and TN. This visualization aids in identifying frequently misclassified classes, reveals confusion patterns, and enables the assessment of the minority class model’s performance, a crucial aspect when dealing with imbalanced datasets.

### Dataset description

This paper applies its methodology to two different public datasets to evaluate the performance of the proposed model. The first dataset contains seven classes, representing various tomato leaf diseases and also healthy leaves. The second dataset includes nine classes, providing a more diverse set of conditions and challenges for classification. This diversity in datasets is utilized to assess the model’s generalization ability and its accuracy in distinguishing between multiple disease types under varying class distributions.

#### Taiwan tomato leaf disease dataset (Taiwan DS)

This dataset is a detailed and diverse tomato leaf disease images obtained from the Roboflow platform^37^. There are 10047 annotated images, categorized into nine distinct classes: eight common tomato leaf diseases–EB, LB, LMI, LM, MV, SLS, SM, and YLCV, as well as a class representing healthy tomato leaves. The tomato leaf images were gathered under multiple conditions to verify high realism and variability. Several factors influenced leaf images, including leaf occlusion, sunlight intensity, and complex natural backgrounds. The dataset annotations were provided in YOLO object detection format, with class labels and corresponding bounding box coordinates for infected regions. As well as, this dataset was divided into train, test, and valid subsets to ensure reliable model training and performance evaluation.

**Fig. 5:**
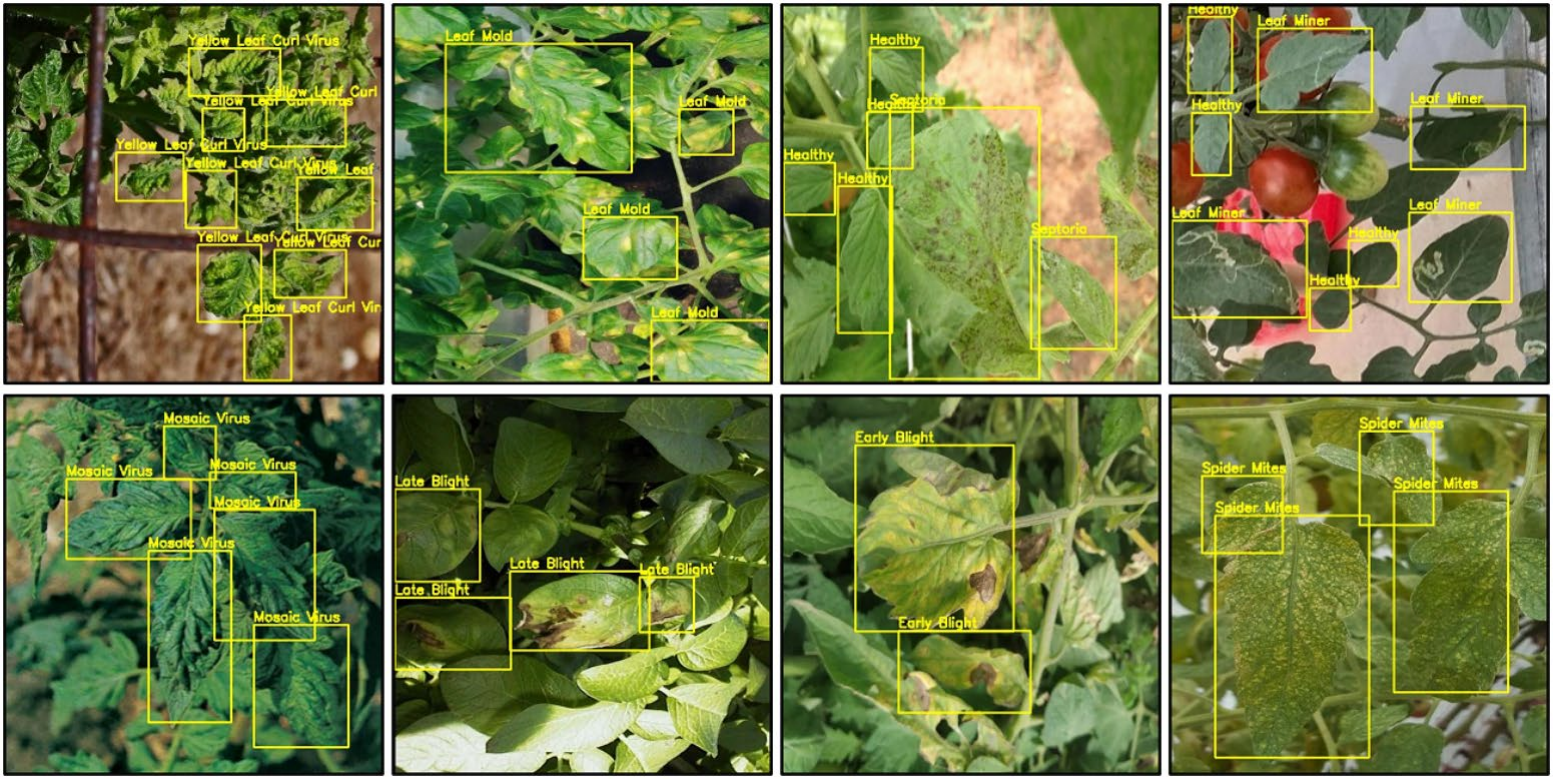
Some samples of Taiwan tomato leaf disease dataset.

Each leaf image in this dataset is 512 *×* 512 pixels in consistent resolution, ensuring consistency throughout model training and evaluation. Moreover, this dataset represents a significant variability in terms of disease symptoms, encompassing various stages of infection and a broad spectrum of lesion characteristics, including shape, color, size, and texture. This diversity enables it to generate disease detection models that are generalizable. Some of these images show leaves with individual diseases, and other images show leaves with more than one disease. Overall, this dataset offers a rich resource for advancing automated plant disease recognition systems. Figure 5 presents some representative samples of this dataset. Table 5 indicates the summary of this dataset.

**Table 5:** The summary of Taiwan dataset images.

**Class**	**Train**		**Valid**		**Test**	
	**Images**	**Instances**	**Images**	**Instances**	**Images**	**Instances**
all	9039	33866	843	2680	165	567
EB	2097	3141	214	252	57	72
HE	783	3336	76	277	19	68
LB	2835	4264	238	344	56	72
LMI	2012	3171	199	259	46	56
LM	2373	4186	211	338	54	87
MV	2902	4057	250	319	57	77
SE	2218	3888	203	324	37	41
SM	1716	2721	123	252	35	44
YLCV	1672	5102	152	315	29	50

#### Tomato leaf diseases detection computer vision dataset (TLDDCV)

**Fig. 6:**
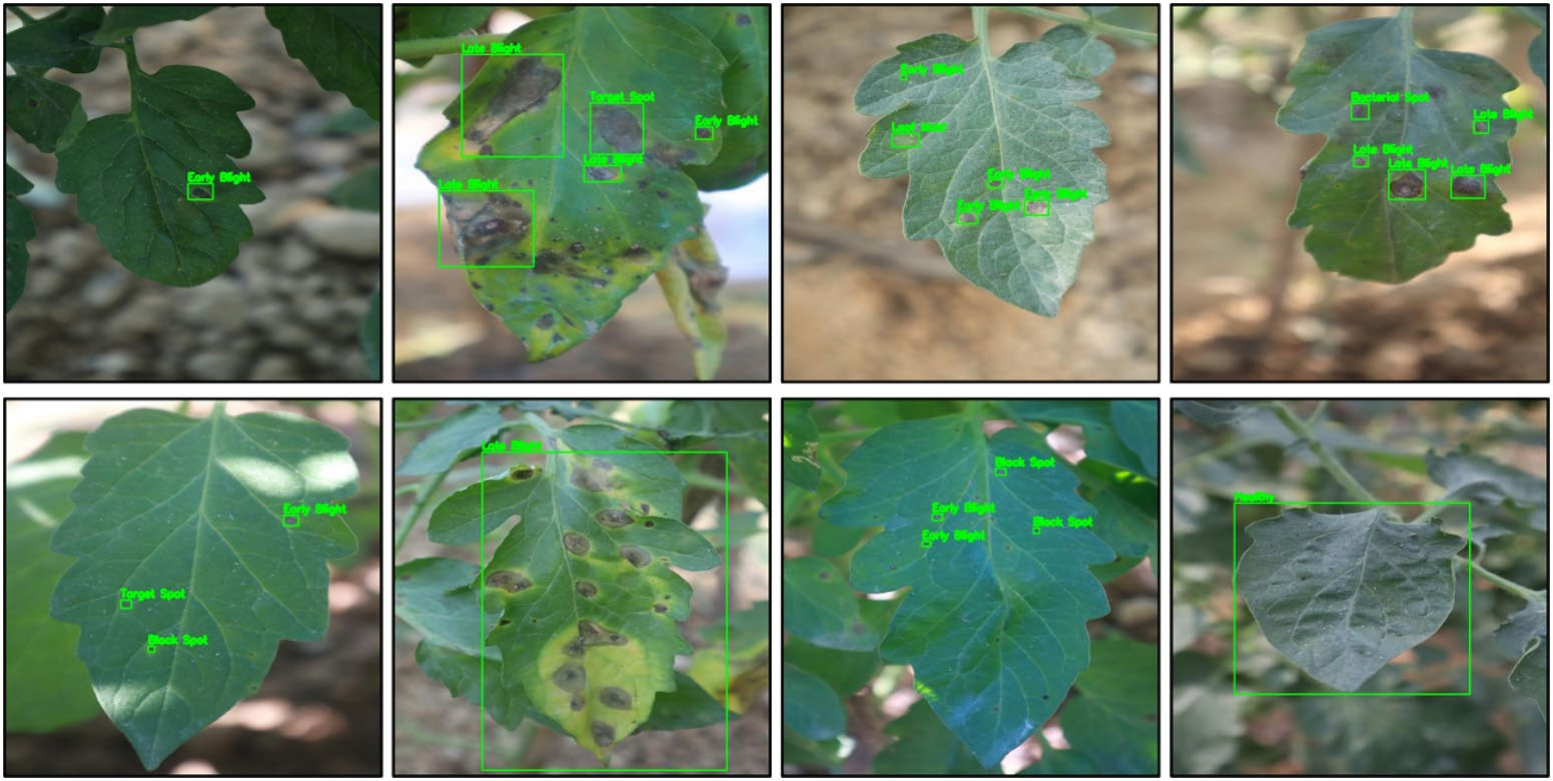
Some samples of tomato leaf diseases detection computer vision dataset.

The dataset comprises seven categories of tomato leaf diseases^36^: *BS*, *EB*, *HE*, *LB*, *LM*, *TS*, and *S*. To handle the limitation of data diversity, image augmentation techniques have been applied to each image. In total, this dataset comprises 737 images, with 645 images allocated to the training set, 31 to the testing set, and 61 to the validation set. The dataset annotations were provided in YOLO object detection format, including class labels and bounding box coordinates for diseased regions. The dataset images were collected under different environmental conditions with variations in illumination, leaf orientation, and background complexity in addition, this dataset was divided into train, test, and valid subsets to ensure reliable model training and performance evaluation. Table 6 presents the summary of this dataset. As illustrated in Fig. 6, the dataset presents two primary challenges:

1. **Small-scale symptoms:** The signs of tomato leaf diseases are often very small, sometimes barely noticeable to the human eye. As shown in Fig. 6, the affected regions typically occupy less than 10% of the entire image area.
2. **Co-occurring symptoms:** It is common for a single leaf to exhibit multiple disease symptoms simultaneously. Moreover, the similarity among various leaf symptoms causes complexity, making it more difficult to correctly differentiate between different disease types.

**Table 6:** The summary of the TLDDCV dataset images.

**Class**	**Train**		**Valid**		**Test**	
	**Images**	**Instances**	**Images**	**Instances**	**Images**	**Instances**
all	645	2124	61	196	31	119
Bacterial Spot	45	84	3	4	2	2
Early Blight	321	1090	31	96	15	66
Healthy	162	162	19	19	7	7
Late Blight	144	291	13	29	9	20
Leaf Mold	45	87	4	7	4	6
Target Spot	30	39	5	6	2	4
Black Spot	191	371	21	35	9	14

## Experimental results and discussion

This section presents a detailed analysis of the experiments evaluated on the selected datasets. This evaluation focuses on three key aspects to assess the effectiveness of the techniques employed. Firstly, the comparisons of applying many lightweight YOLO versions on the Taiwan DS dataset. Secondly, the experimental comparisons of improving the YOLOv11n backbone with various backbone networks. Finally, we compare the proposed methodology with previous research approaches to highlight its advantages and improvements. Each comparison intends to shed light on the best design decision choices for accurate and efficient object detection.

### Using various versions of YOLO architectures on Taiwan DS

Algorithms for real-time object detection, such as the YOLO family, have become fundamental in various tasks of computer vision because of their ability to perform accurate and fast detection in a single forward pass^17,56,57^. These models are widely used in applications where speed is crucial, such as autonomous driving, surveillance, and robotics. In this subsection, we focus on applying and evaluating several lightweight versions of the YOLO architecture to determine which version provides the better balance between computational efficiency and detection accuracy. The goal is to assess their performance on the Taiwan DS dataset and determine which model is best suited for deployment in real-time, resource-constrained applications.

Table 7 presents a comparative evaluation of multiple YOLO versions on the Taiwan DS dataset. Each model was trained for 100 epochs, and its performance was assessed using recall, precision, F1-score, mAP50, and mAP50-95 metrics. The obtained results are summarized as follows:

- The YOLOv8n^58^ is the first invented version under the new Ultralytics framework, representing a strong architecture with improved trade-offs between accuracy and speed. It achieved good results, especially in mAP50, with a value of 96.6%, and a competitive F1-score of 91.8%.
- The YOLOv9t^59^ combines structural reparameterization techniques and upgraded neck designs, such as RepNeck and SimSPPF, to enhance inference-time efficiency. While achieving a high precision of 92.3%, it also demonstrated solid generalization capabilities with an F1-score of 91.25%.
- The YOLOv10n^60^ ensured further architectural simplification and decreased computational costs while achieving competitive performance. Despite a slightly lower F1-score (88.72%), it became efficient for lightweight real-time deployment.
- The YOLOv11n^61^ presented significant enhancements in feature aggregation and context-awareness by combining Transformer-based components and attention mechanisms. As a result, it outperforms all previous versions, achieving the highest precision (95.4%), recall (94.4%), and F1-score (94.8%), making it the best-performing model on the Taiwan DS dataset.

In summary, YOLOv11n achieved superior performance compared to other YOLO models, ensuring its suitability and robustness for high-accuracy detection tasks on the Taiwan DS dataset.

**Table 7:** The comparison between multiple versions of YOLO architectures on Taiwan DS.

Algorithm	Epochs	Precision	Recall	F1-Score	mAP50	mAP50-95
YOLOV8n	100	94.2	89.6	91.8	96.6	88.6
YOLOV9t	100	92.3	90.2	91.25	96.3	89.1
YOLOV10n	100	90.3	87.2	88.72	94.6	87
YOLOV11n	100	95.4	94.4	94.8	97.6	93

### Using different backbones in YOLO11N architecture

In this subsection, we examine the effect of modifying the backbone within the YOLOv11n architecture. The aim is to assess how various feature extractor techniques affect the model’s performance on both datasets tested in this paper. Particularly, many transformer-based backbones have been integrated to examine their effects on detection accuracy and feature selection. Transformer-based architectures, in particular, have demonstrated a strong ability to capture global contextual information, which can enhance the model’s focus on the most relevant features during object detection.

#### Evaluation on Taiwan dataset

To explore the effect of various transformer-based backbones on object detection performance, we integrated several related transformer architectures into the YOLO11n framework and evaluated them on the Taiwan DS dataset. As presented in Table 8, the baseline YOLO11n model already proved strong performance, but the introduction of transformer backbones led to notable variations in precision, recall, F1-Score, mAP50, and mAP50-95 quality metrics.

Among all the evaluated models, the integration of YOLO11n with DeiT achieves the highest results, with a precision of 97.3%, an F1-Score of 95.5%, and a mAP50-95 of 93.4%. The DeiT model was presented by Touvron et al.^50^ as a lightweight vision transformer that utilizes knowledge distillation to achieve high accuracy with decreased computational costs and training data. Its efficient attention mechanism helps in better global feature extraction.

The Swin Transformer, introduced by Liu et al.^62^, is a hierarchical transformer that calculates attention within shifted windows, enabling better scalability and local modeling. In our experiments, Swin demonstrated competitive precision (95.9%) but suffered from a drop in recall and mAP50-95 (88.9%), possibly due to limitations in handling fine object details within its local window structure.

MobileViT, designed by Mehta and Rastegari^63^, combines convolutional layers with transformers to make mobile-friendly architectures. It introduces a robust balance between efficiency and performance, achieving an F1-Score of 94% and mAP50-95 of 90%, making it suitable for use with edge devices.

CrossViT, presented by Chen et al.^64^, provides a dual-branch architecture that operates various image scales in parallel and combines them using cross-attention. It can successfully extract both local and global context thanks to its design. In our results, CrossViT achieved a high precision (96.7%) and a strong mAP50-95 (92.8%), indicating its robustness across multi-scale features. EfficientFormer-l1, introduced by Li et al.^65^, concentrates on speed and low memory usage, and is developed for real-time inference on devices with limited resources. While it achieved an acceptable precision (93.9%), it had the lowest mAP50-95 (87.9%) among the tested models, suggesting that its lightweight design may sacrifice some representational depth.

MaxViT, presented by Tu et al.^66^, combines block-wise and grid attention to model both short- and long-range dependencies. Despite showing high precision (95.1%), it underperformed in recall (89.9%) and mAP50-95 (86.3%), which may be due to its complex attention mechanism not fully aligning with the object scales and patterns in the DS2 dataset. In summary, the experimental results clearly ensure that DeiT is the most effective backbone for the YOLO11n architecture, offering superior accuracy and generalization. Its simple yet powerful transformer design provides a decent trade-off between computational efficiency and detection performance.

**Table 8:** The comparison of YOLO11n with transformers modifying on Taiwan DS.

Algorithm	Epochs	Precision	Recall	F1-Score	mAP50	mAP50-95
YOLOV11n	100	95.4	94.4	94.8	97.6	93
YOLOV11n&Deit	100	97.3	93.8	95.5	97.8	93.4
YOLOV11n&Swin	100	95.9	92.4	94.1	97.3	88.9
YOLOV11n&MobileViT	100	95.4	92.6	94	97.6	90
YOLOV11n&CrossViT	100	96.7	92.7	94.66	97.9	92.8
YOLOV11n&EfficientFormer_l1	100	93.9	92.3	93.1	97.5	87.9
YOLOV11n&MaxViT	100	95.1	89.9	92.42	96.3	86.3

We conducted a statistical significance analysis using McNemar’s test to compare YOLOv11 and the proposed YOLOv11–DeiT model. The test results showed no statistically significant difference between the two models (p-value = 1.0). However, the obtained performance metrics, including precision, recall, and F1-score, consistently demonstrate the effectiveness and robustness of the proposed YOLOv11–DeiT model. This indicates that the proposed model achieves competitive and stable performance across evaluation measures. The lack of statistical significance can be attributed to the very small number of disagreement cases between the two models, which limits the ability of the statistical test to capture meaningful differences.

**Fig. 7:**
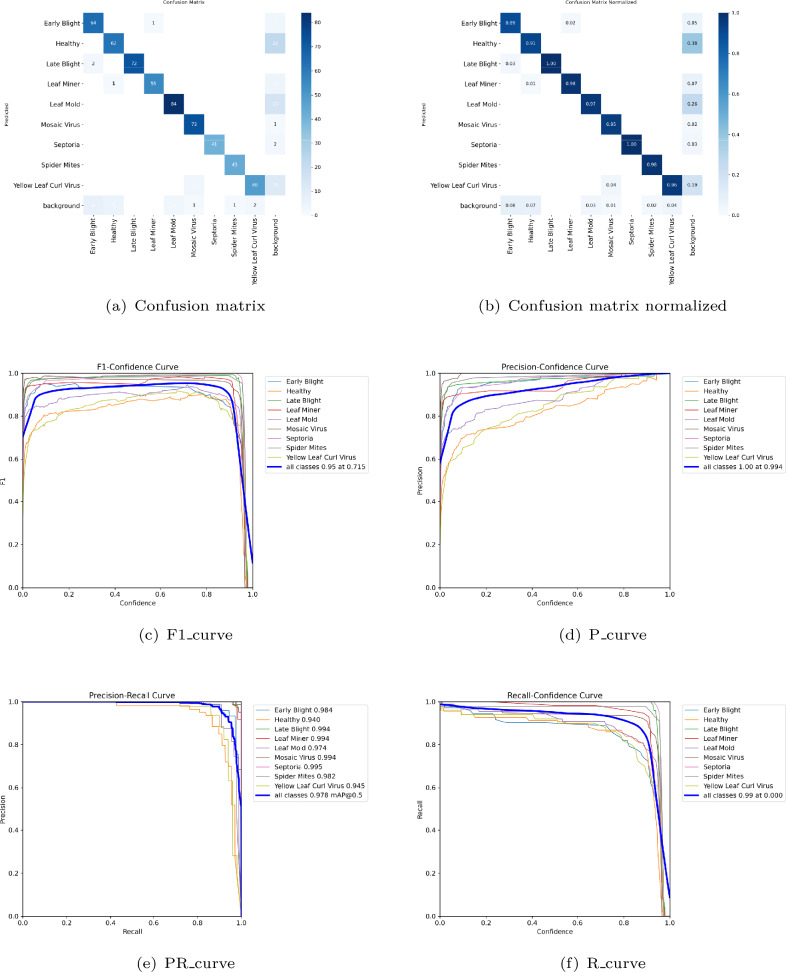
Visualization of classification results on Taiwan test images and its metric curves.

Figure 7 illustrates the visualizations that represent the performance enhancement of the proposed classification model. The first row shows the confusion matrix and its normalized version, illustrating the relationships between true and predicted labels. The normalized matrix provides a clearer view of the system’s performance across all classes by representing the values as percentages. The middle and bottom rows comprise numerous performance curves. The F1_curve strengthens the trade-off between precision and recall. At the same time, the precision curve (P_curve) and recall curve (R_curve) provide detailed insights into the model’s performance, enabling it to correctly detect positive cases and return related instances, respectively. The trade-off between recall and precision at multiple threshold values is illustrated by the Precision-Recall curve (PR curve).

**Fig. 8:**
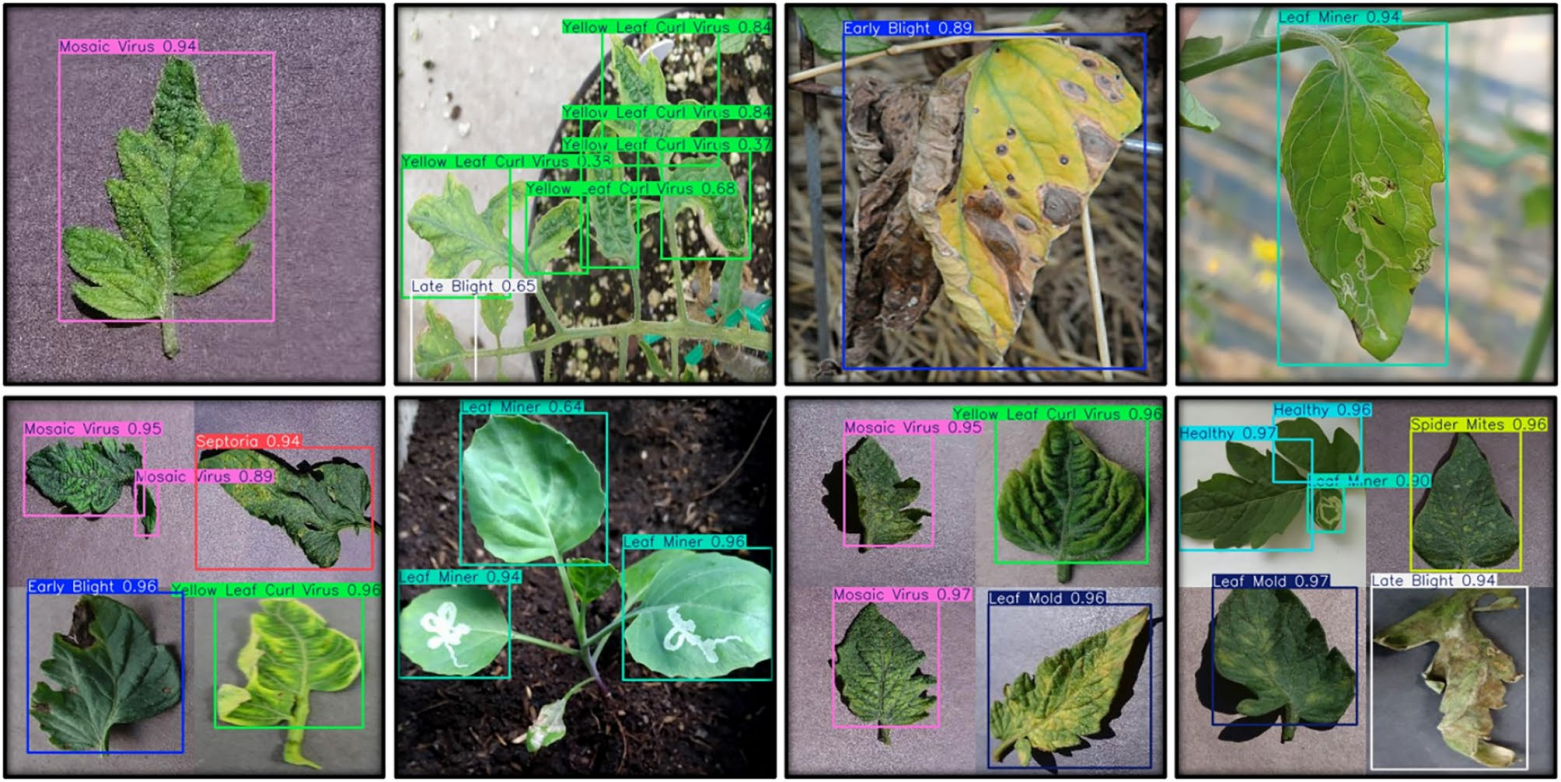
The visual results of localization and classification on leaf disease test images in Taiwan DS.

Figure 8 displays the qualitative results of the proposed framework for detecting tomato leaf diseases, applied to various leaf images. Each sub-image indicates one or more leaves annotated with bounding boxes that classify the detected disease classes. These bounding boxes have been labeled with related disease names, demonstrating the system’s ability to recognize and detect various leaf disease types in multiple plant species. The diversity of leaf backgrounds, shapes, and disease symptoms highlights the system’s robustness in handling real-world variability. These visual cases ensure the effectiveness of the identification system, especially in disease-prone regions, which is essential for assisting early diagnosis applications and precision agriculture.

As well known preprocessing techniques have an important role in improving the images quality and deep learning robustness models. Especially, applying bilateral filter and gamma correction helps in reducing noise, improving contrast, and preserving significant structural details, which leads to better feature extraction and discrimination between healthy and diseased regions. Table 9, indicates the experimental results of the proposed methodology with and without processing step. Specifically, the precision increased from 96.2% to 97.3%, while the recall showed a slight improvement from 93.4% to 93.8%. Consequently, the F1-score improved from 94.74% to 95.5%, indicating a better balance between precision and recall. Furthermore, the mAP50 and mAP50-95 metrics also experienced enhancements, rising from 97.5% to 97.8% and from 92.6% to 93.4%, respectively. These results clearly demonstrate that incorporating preprocessing steps contributes to improving the detection accuracy and generalization capability of the proposed model.

**Table 9:** Quantitative Analysis of the Impact of Preprocessing step on Model Performance on Taiwan DS.

Algorithm	Epochs	Precision	Recall	F1-Score	mAP50	mAP50-95
YOLOV11n&Deit without preprocessing	100	96.2	93.4	94.74	97.5	92.6
YOLOV11n&Deit with preprocessing	100	97.3	93.8	95.5	97.8	93.4

In real-world applications, both detection performance and computational efficiency are important factors. Therefore, we carefully examined how different batch sizes affect the inference performance of the proposed YOLOv11n-DeiT model. We evaluated batch sizes of 8, 16, and 32 to see how they influenced detection accuracy and inference time.

The experimental results show that increasing the batch size improves computational efficiency by decreasing the overall inference time per image. However, very large batch sizes can slightly reduce detection performance because of memory limits and processing overhead. Among the configurations tested, a batch size of 16 provided the best balance between detection performance and inference speed.

Table 10 offers a detailed comparison of performance metrics and inference time for different batch sizes. The results indicate that the proposed model maintains high detection accuracy while achieving efficient inference. This makes it suitable for real-world deployment scenarios where both speed and accuracy are crucial.

**Table 10:** Impact of different batch sizes on detection performance and inference time on Taiwan DS.

**Batch Size**	**Precision**	**Recall**	**F1-Score**	**mAP50**	**mAP50-95**	**Inference Time**
8	96.3	93.2	94.7	97.5	92.6	191.45 ms per image
16	97.3	93.8	95.5	97.8	93.4	184.81 ms per image
32	95.6	92.8	94.18	97.2	91.5	182.54 ms per image

The ablation study results demonstrate that each component of the proposed framework contributes to the overall detection performance. To provide a clearer interpretation of the contribution of each module, an additional “Evaluated Component” column has been incorporated into Table 11, explicitly identifying the component assessed in each experimental configuration. The reported results were obtained after extensive experimental trials conducted under consistent training settings to ensure reliability and reproducibility.

The DeiT module enhances feature extraction and representation capabilities, while the preprocessing stage improves image quality and facilitates the extraction of disease-related characteristics. Furthermore, the genetic optimization strategy contributes to improving the overall detection performance through more effective parameter selection. The best performance was achieved by the complete proposed framework, which attained an F1-score of 95.5%, a mAP50 of 97.8%, and a mAP50-95 of 93.4% on the Taiwan DS dataset. Table 11 presents the component wise ablation study and highlights the contribution of each module to the final performance.

**Table 11:** Component-wise Ablation Study of the Proposed Framework on Taiwan DS.

Algorithm	Evaluated Component	Epochs	Precision	Recall	F1-Score	mAP50	mAP50-95
YOLOV11n without preprocessing	Baseline	100	95.4	94.4	94.8	97.6	93
YOLOV11n&DeiT without preprocessing	DeiT Module	100	96.2	93.4	94.74	97.5	92.6
YOLOV11n&DeiT without genetic	Genetic Algorithm	100	96	93	94	97.5	92.5
YOLOV11n&DeiT with preprocessing	Image Preprocessing	100	97.3	93.8	95.5	97.8	93.4

The suggested YOLOv11–DeiT framework was tested on the Taiwan dataset through several independent experiments for the purpose of ensuring the reliability and stability of the achieved results. As can be seen from Table 12, the suggested framework was performed five times under identical conditions, with all the indicators of its efficiency remaining at an extremely high level for all runs. The achieved Average Precision, Recall, F1-score, mAP50, and mAP50–95 equalled 97.30%, 93.80%, 95.52%, 97.84%, and 93.40%, respectively, with the standard deviations being quite small. It indicates that the achieved results were highly reliable and consistent, thereby proving the effectiveness of the suggested YOLOv11–DeiT framework.

**Table 12:** Statistical results (Mean ± standard deviation) across repeated runs Taiwan ds.

**Dataset**	**Run**	**Precision**	**Recall**	**F1-score**	**mAP50**	**mAP50-95**
**Taiwan ds**	1	96.60	93.40	94.97	98.00	92.90
	2	97.30	93.20	95.21	97.70	93.40
	3	97.40	94.00	95.67	97.80	93.50
	4	97.80	94.20	95.97	97.90	93.60
	5	97.40	94.20	95.77	97.80	93.60
	**Avg**	**97.30**	**93.80**	**95.52**	**97.84**	**93.40**
	**Std**	0.39	0.42	0.37	0.10	0.26

#### Evaluation on TLDDCV dataset

To further confirm the strength and generalization ability of the proposed framework, the same experimental settings and evaluation procedures were applied to another dataset. This aimed to show that the proposed model can consistently perform well across various datasets and is not restricted to a certain data distribution.

The experimental results in Tables 13 and 14 show how effective the proposed framework is on the TLDDCV dataset. In the first experiment, we evaluated multiple YOLO architectures, including YOLOV8n, YOLOV9t, YOLOV10n, and YOLOV11n, to find the best detection model. The results show that YOLOV11n achieved the best overall performance, with the highest F1-Score of 71.39%, mAP50 of 72.8%, and mAP50-95 of 47.5%. This confirms its superior ability to detect plant diseases compared to the other YOLO versions.

In the second experiment, we combined the YOLOV11n architecture with several transformer-based models, including DeiT, Swin, MobileViT, CrossViT, EfficientFormer_l1, and MaxViT, to further improve feature representation and detection performance. Among all transformer combinations, the YOLOV11n based on DeiT model produced the best results, with an F1-Score of 76.4%, mAP50 of 73%, and mAP50-95 of 48%. These findings show that combining DeiT with YOLOV11n greatly enhances the model’s ability to capture important disease features and improves overall detection accuracy on the TLDDCV dataset.

**Table 13:** The comparison between multiple versions of YOLO architectures on TLDDCV DS.

Algorithm	Epochs	Precision	Recall	F1-Score	mAP50	mAP50-95
YOLOV8n	100	65.9	77	71.05	70.2	44.1
YOLOV9t	100	70.3	63.8	63.8	72.6	46
YOLOV10n	100	65.9	60.6	63.14	69.1	44.5
YOLOV11n	100	70.5	72.3	71.39	72.8	47.5

**Table 14:** The comparison of YOLO11n with transformers modifying on TLDDCV DS.

Algorithm	Epochs	Precision	Recall	F1-Score	mAP50	mAP50-95
YOLOV11n	100	70.5	72.3	71.39	72.8	47.5
YOLOV11n&Deit	100	83.9	70.3	76.4	73	48
YOLOV11n&Swin	100	67.9	60	63.71	65.8	39.1
YOLOV11n&MobileViT	100	69.7	66	67.8	65.7	42.9
YOLOV11n&CrossViT	100	67.2	73.6	70.3	72.1	46.1
YOLOV11n&EfficientFormer_l1	100	44.1	56.4	49.5	51.9	32.8
YOLOV11n&MaxViT	100	54.7	52.8	53.8	43.6	28.1

**Fig. 9:**
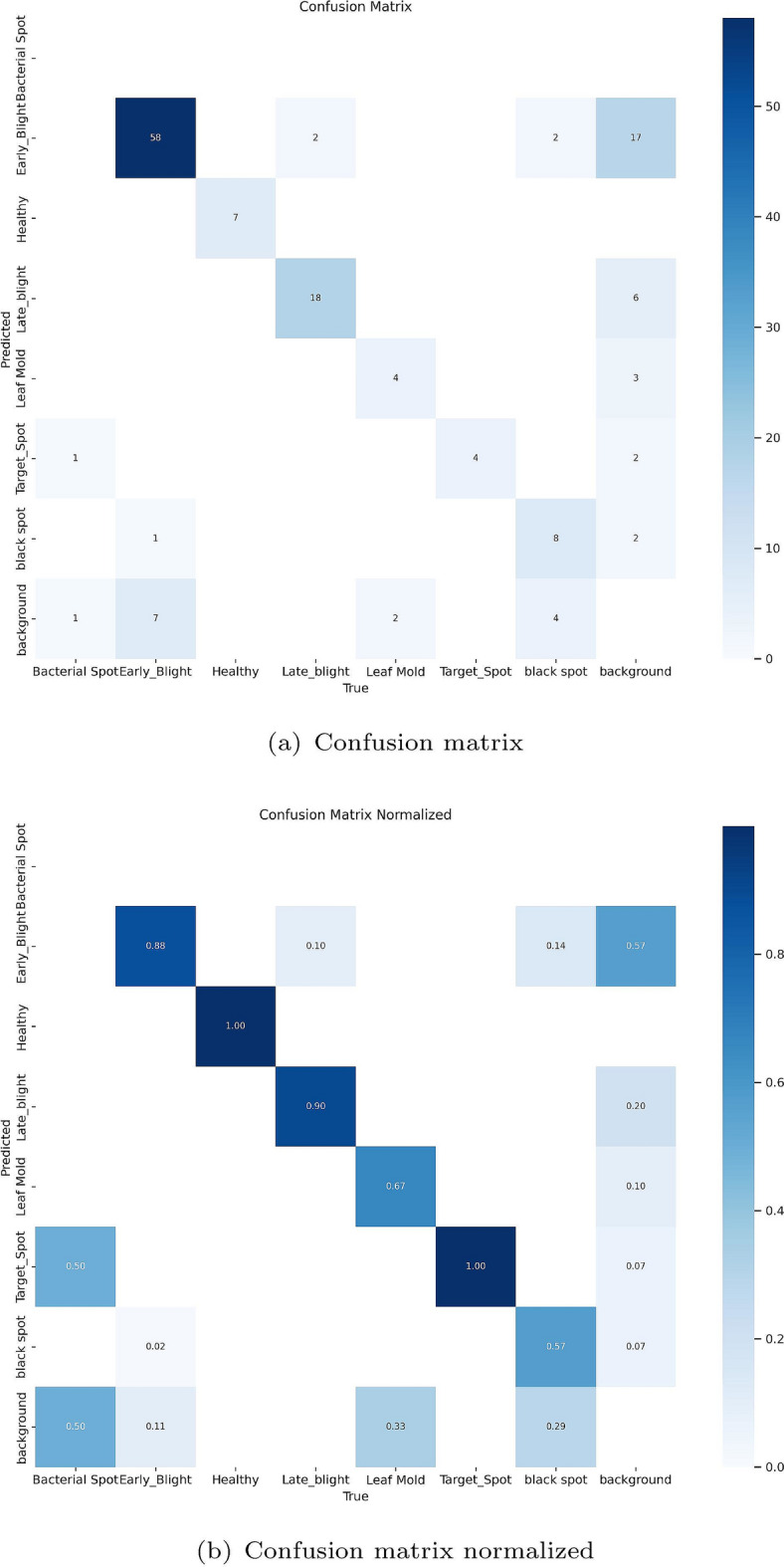
Confusion matrices of classification results on TLDDCV test images.

**Fig. 10:**
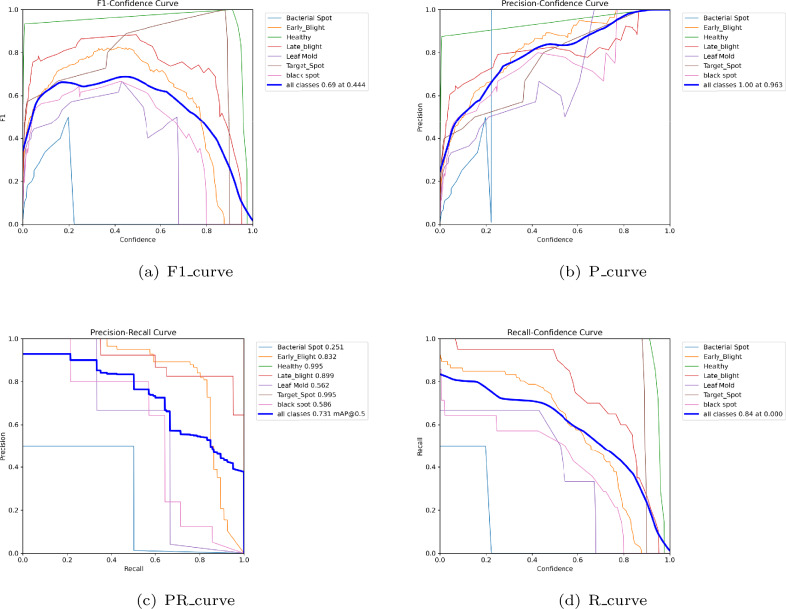
visualization of classification results on TLDDCV test images and metric curves.

**Fig. 11:**
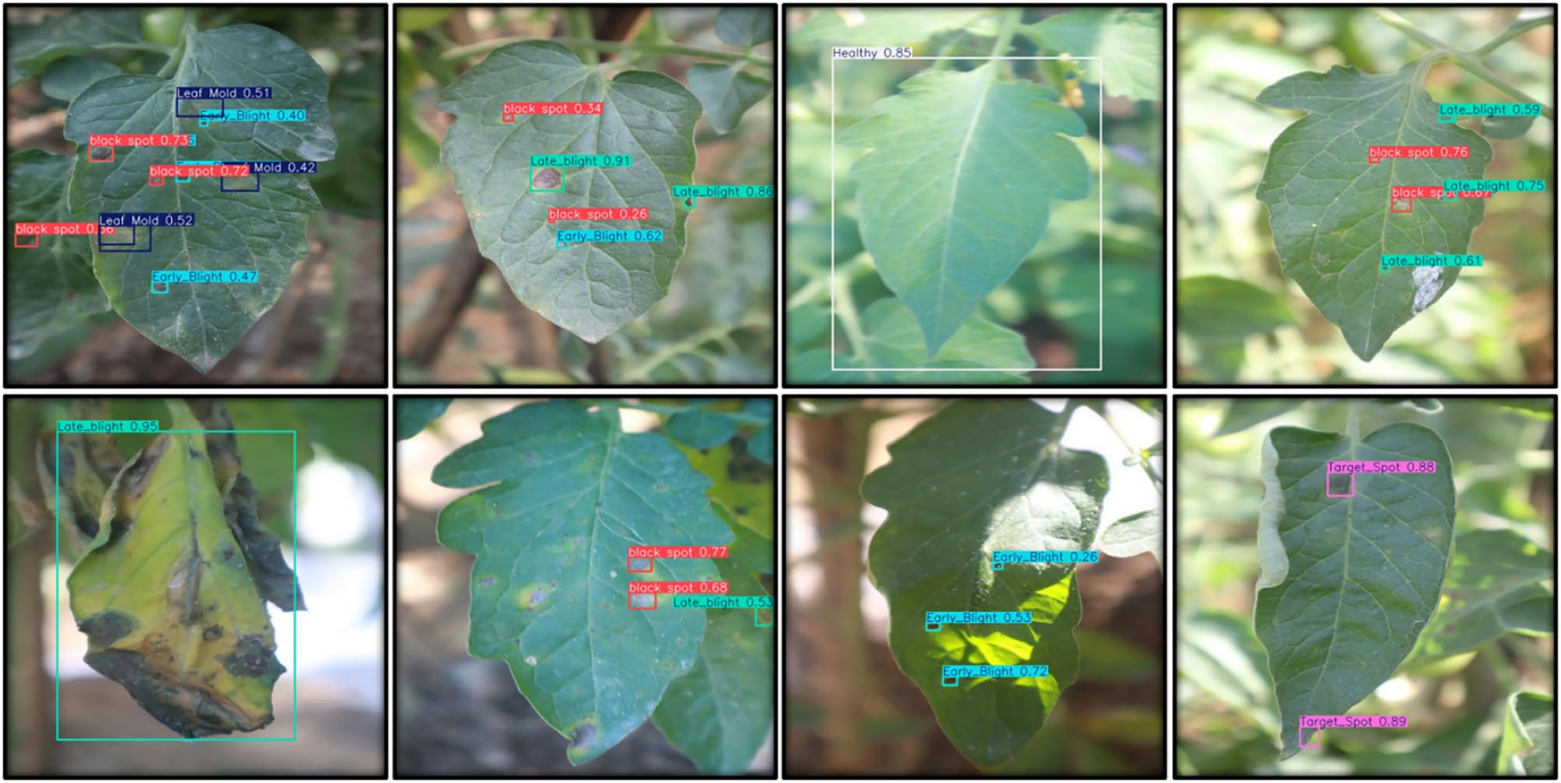
The visual results of localization and classification on leaf disease test images in TLDDCV DS.

In this subsection, many transformer-based backbones have been used within the YOLO11n model to improve the feature extraction effectiveness in detecting tomato plant leaf diseases. These trials have been applied to the Taiwan dataset to determine the optimal configuration. Among all evaluated configurations, the YOLO11n equipped with the DeiT transformer backbone delivered the highest performance, achieving superior results in multiple metrics, including precision and detection accuracy. Based on these findings, the best-performing model configuration has been directly applied to the TLDDCV DS dataset to further assess its generalization capability and robustness across various data conditions. Figures 9 and 10 indicate the visualization of applying the proposed methodology on the TLDDCV dataset (Fig. 11).

The results shown in Table 15 illustrate the strong effect of the preprocessing step on the performance of the proposed YOLOV11n&DeiT-based model. We see that using image preprocessing improves the model’s classification ability, especially regarding Precision and F1-score. Specifically, the model with preprocessing achieves a Precision of 83.9%, compared to 78% without preprocessing. This indicates a reduction in false positive predictions. Additionally, the F1-score rises from 72.5% to 76.4%, showing a better balance between Precision and Recall. While there is a small drop in Recall and mAP50, the overall enhancement in the key evaluation metrics confirms that preprocessing improves the model’s robustness and ability to distinguish between classes. This emphasizes the need to include preprocessing techniques as an important step to boost detection performance on the TLDDCV dataset.

**Table 15:** Quantitative Analysis of the Impact of Preprocessing step on Model Performance on TLDDCV DS.

Algorithm	Epochs	Precision	Recall	F1-Score	mAP50	mAP50-95
YOLOV11n&Deit without preprocessing	100	78	72.5	72.5	77.1	48.6
YOLOV11n&Deit with preprocessing	100	83.9	70.3	76.4	73	48

The results in Table 16 show how different batch sizes affect model performance. Batch size 16 achieves the best overall results compared to 8 and 32. It produces the highest Precision (83.9%) and F1-score (76.4%), along with the lowest inference time (0.015 ms per image). Though batch size 8 has a slightly higher Recall and batch size 32 demonstrates a similar mAP50, batch size 16 offers the best balance between accuracy and speed. Thus, batch size 16 is the optimal setting for the proposed model.

**Table 16:** Impact of different batch sizes on detection performance and inference time on TLDDCV DS.

**Batch Size**	**Precision**	**Recall**	**F1-Score**	**mAP50**	**mAP50-95**	**Inference Time**
8	70.2	76.7	73.31	74	47.6	0.019 ms per image
16	83.9	70.3	76.4	73	48	0.015 ms per image
32	80.2	69.6	74.58	74.4	44.9	0.02 ms per image

The proposed YOLOv11-DeiT algorithm was assessed using the TLDDCV dataset during five different tests to test its stability. The results presented in Table 17 show that the proposed algorithm has an average Precision score of 83.94%, Recall of 70.28%, F1-score of 76.48%, mAP50 score of 73.24%, and mAP50−95 score of 47.98%. Though the TLDDCV dataset is more complex, the consistency of the results can be observed from.

**Table 17:** Statistical results (Mean ± standard deviation) across repeated runs on TLDDCV ds.

**Dataset**	**Run**	**Precision**	**Recall**	**F1-score**	**mAP50**	**mAP50-95**
**TLDDCV**	1	84.30	71.80	77.55	73.20	47.70
	2	83.50	69.50	75.87	75.20	47.80
	3	83.80	70.60	76.50	72.10	48.40
	4	83.90	70.30	76.50	73.00	48.00
	5	84.20	69.20	75.97	72.70	48.00
	**Avg**	**83.94**	**70.28**	**76.48**	**73.24**	**47.98**
	**Std**	0.29	0.92	0.60	1.05	0.24

### The comparison based on data splitting methods

Data splitting plays an important role in evaluating how well machine learning models perform and generalize. This study uses two common data splitting strategies: the holdout method and cross-validation. The holdout method separates the dataset into two sets: a training set and a testing set. The model is trained on the training set and evaluated on the separate testing data. While this method is straightforward and efficient, its performance can vary based on how the data is divided. In contrast, cross-validation offers a more dependable evaluation by splitting the dataset into several folds. This study uses 5-fold cross-validation, where the dataset is divided into five equal parts. The model is trained on four parts and tested on the remaining one. This process repeats until each part has been tested. The final performance is calculated by averaging the results from all folds, which helps reduce overfitting and provides a stronger estimate. Both methods are used in this study to ensure a thorough evaluation of the proposed model. The results from each fold in cross-validation, along with the holdout results, are shown in Table 18. The consistent performance across all folds demonstrates the robustness and stability of the proposed model. Moreover, the small gap between training and testing results indicates good generalization capability with no significant overfitting. The results from the second dataset (TLDDCV DS), shown in Table 19, further confirm the reliability and steady performance of the proposed model in both holdout and 5-fold cross-validation settings.

**Table 18:** Performance evaluation using 5-fold cross-validation and holdout method across training, validation, and testing sets on Tiawan DS.

**Method**	**Fold**	**Set**	**Precision**	**Recall**	**F1-Score**	**mAP50**	**mAP50-95**
Cross Validation	Fold 1	Train	98	98.2	98.1	99.3	94.4
		Validation	95.7	93.2	94.4	97.4	90.8
		Test	95.7	92.8	94.2	97.5	91
	Fold 2	Train	96.6	95.6	96.1	98.6	91.9
		Validation	93.9	92.5	93.2	96.8	89
		Test	94.2	94.5	94.3	98	90.4
	Fold 3	Train	96.1	95.6	95.8	98.6	91.9
		Validation	94.3	92.9	93.6	97.6	90.1
		Test	92.5	95.2	93.3	97.9	90.4
	Fold 4	Train	96.4	95.5	96	98.3	91.8
		Validation	93.5	92.3	93	96.4	89
		Test	94.2	94.5	94.2	98	90.4
	Fold 5	Train	97.9	98.1	98.1	99.2	94.5
		Validation	94.5	93.4	94	97.3	90.7
		Test	96.1	94.6	95.4	98.5	92.4
5-Fold CV result (Average Test)	—	—	94.56	94.02	94.28	97.98	91.32
Holdout	–	Train	95.1	92.5	93.9	97.3	91.8
		Validation	94.8	92.5	93.7	97.5	92
		Test	97.3	93.8	95.5	97.8	93.4

**Table 19:** Performance evaluation using 5-fold cross-validation and holdout method across training, validation, and testing sets on TLDDCV DS.

**Method**	**Fold**	**Set**	**Precision**	**Recall**	**F1-Score**	**mAP50**	**mAP50-95**
Cross Validation	Fold 1	Train	85.4	86	85.7	90.9	50.2
		Validation	80	80.4	80.2	72.1	48.1
		Test	80.3	82.5	81.4	75.5	47.6
	Fold 2	Train	80.6	80.8	80.7	70.6	46.2
		Validation	72.1	78	74.9	75.3	48.1
		Test	77.9	75.9	76.9	75.9	45
	Fold 3	Train	77.3	76.6	76.9	77	50.7
		Validation	77.8	80.3	79.0	76.9	47.3
		Test	72.5	76.6	74.5	77.2	46.2
	Fold 4	Train	78.7	75.8	77.2	72.1	48.6
		Validation	75.7	76.6	76.1	78.5	45.9
		Test	79	74.3	76.6	75.2	45.4
	Fold 5	Train	79.5	78.5	79.0	72.4	47.1
		Validation	74.3	82.5	78.2	75.8	46.7
		Test	74.3	76.5	75.4	78.8	48.7
5-Fold CV result (Average Test)	—	—	76.8	77.2	77	76.5	46.6
Holdout	–	Train	80.5	74.6	77.4	72	48.2
		Validation	77.1	76	76.5	75.5	49
		Test	83.9	70.3	76.4	73	48

### Grad-CAM visualization after detection

Grad-CAM (Gradient-weighted Class Activation Mapping) is a popular approach for making machine learning models more interpretable as it indicates which parts of an image have the greatest influence on the prediction made by the machine learning model^67–69^. The algorithm computes the gradients of the class that is predicted by the model relative to the last convolution layer in order to generate heatmaps representing the spatial importance of the image regions. The proposed model, specifically YOLOv11 and DeiT-based, utilizes the approach.

**Fig. 12:**
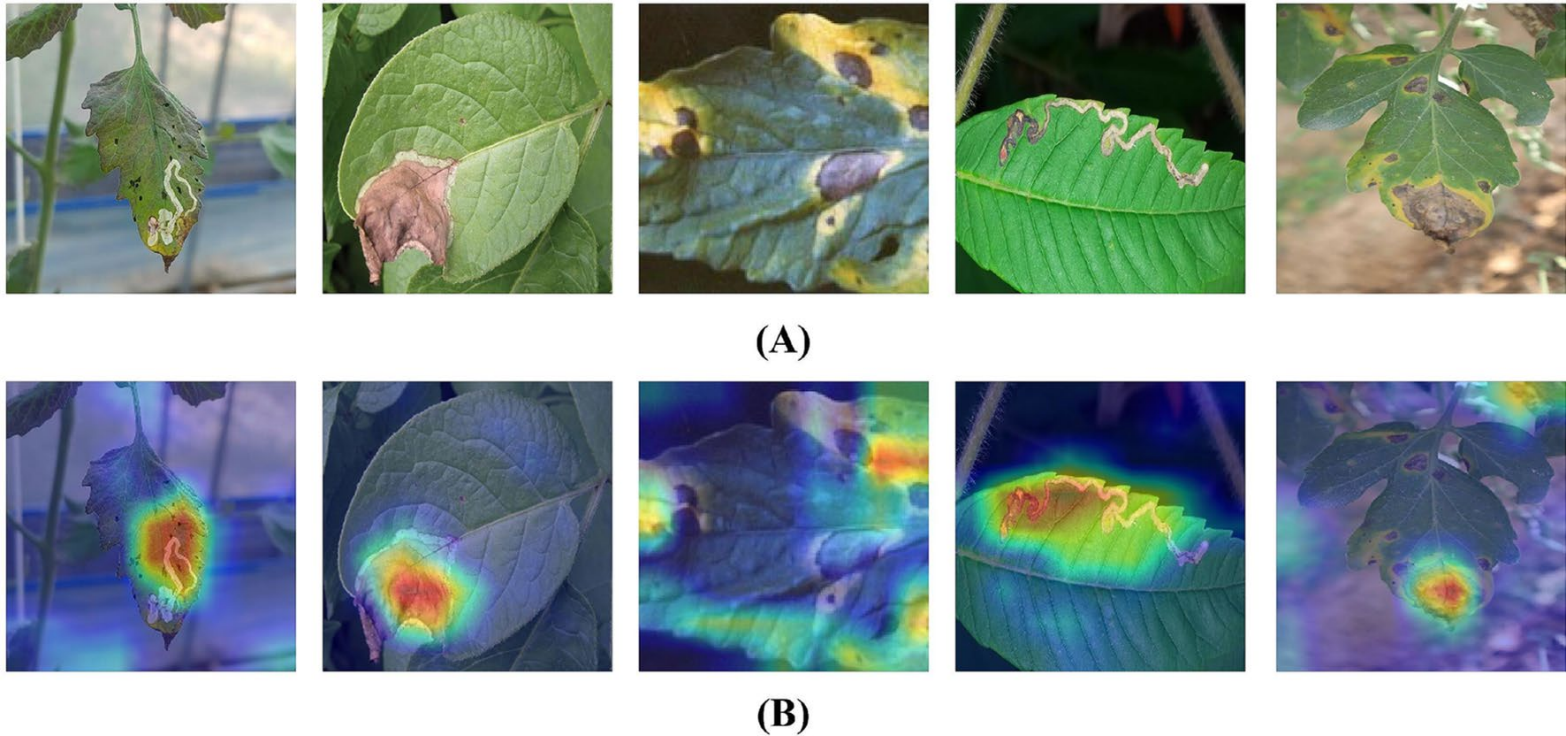
The visual results of localization and classification on leaf disease test images in TLDDCV DS.

As shown in the visualization produced using this method Fig. 12, it can be seen from the heatmap overlay that the model concentrates mainly on the discriminative regions within the picture, as indicated by warm colors such as red and yellow, while the other regions are represented by cooler colors such as blue and green. When comparing the Grad-CAM output with the original image, it is evident that the model focuses on disease-related regions rather than the background, which confirms that the learned features are meaningful and class-relevant.

### The comparison with previous research

This subsection evaluates the effectiveness of the proposed methodology by applying it to both datasets used in this work. The performance results are then compared with previous research works. The purpose is to demonstrate the competitiveness and efficiency of the proposed algorithm in addressing the object detection task. By benchmarking our approach against existing methods, we aim to validate the improvements achieved in terms of accuracy and robustness. To ensure a fair and meaningful comparison, we emphasize that the primary evaluation is conducted against methods that utilize the same benchmark datasets as those used in this study.

The YOLOv11n-DeiT framework not only offers better detection performance than current methods but also has a lightweight architecture that improves feature representation. While adding DeiT boosts the model’s ability to distinguish features, the framework still keeps low computational complexity and fast inference speeds compared to more complex deep learning methods. Additionally, the lightweight design of YOLOv11n helps lower memory use and costs, making it ideal for real-time agricultural applications and for use on devices with limited resources. This approach successfully balances detection accuracy and computational efficiency.

#### Using Taiwan dataset

Table 20 provides a comparison study between the proposed methodology and several previous object detection models applied to the Taiwan DS. Each study utilized the same dataset, allowing for a fair comparison across various performance metrics, including precision, recall, F1-score, and mAP. Abulizi et al.(2025)^34^ proposed an edited YOLOv9 version and achieved a precision of 92.5%, recall of 86.6%, and an F1-score of 89.38%, with a respectable mAP50 of 95.1%. This model had notable enhancements over the baseline YOLOv9, especially in terms of detection accuracy. Bachri et al. (2024)^30^ presented the Faster R-CNN model, a two-stage detector known for its high performance but lower speed. Although some metrics were not reported, the mAP50 value of 87.01% represents a reasonable detection performance, given the complexity of the dataset.

Kohut et al. (2024)^28^ also applied the Faster R-CNN model, enhanced with a ResNet50 backbone. Their method achieved a high mAP50 of 96.5%, though the absence of precision, recall, and F1-score values limits a complete performance comparison. Wang et al. (2024)^29^ utilized the YOLOv6 model, a real-time detector optimized for a balance between accuracy and speed. Their model achieved strong results, with 92.9% precision, 95.2% recall, and 93.8%mAP@500, showcasing excellent generalization.

In contrast, more recently, Dhiab et al. (2025)^70^ implemented YOLOv8 on a selected subset of Taiwan DS dataset. The performance of this framework had a precision score of 81.9%, recall score of 67.6%, F1-score of 74.07%, and mAP50 score of 77.4%. Even though the applicability of YOLO-based detectors for identifying tomato diseases was confirmed by the above study, the performance was relatively low compared to the proposed methodology.

Islam et al. (2026)^71^ proposed an intelligent YOLOv9-based framework for detecting tomato leaf disease. The performance of this framework had a precision score of 95.8%, recall score of 92.4%, F1-score of 94.07%, mAP50 score of 97.0%, and mAP50-95 score of 93.0%. However, even though their performance was quite impressive, the proposed YOLOv11n-DeiT framework outperformed theirs with a precision score of 97.3%,

In contrast, the proposed methodology, which combines YOLOv11n and DeiT architectures, achieves the highest performance results in almost all metrics, including 97.3% precision, 93.8% recall, 95.5% F1-score, 97.8% mAP50, and 93.4% mAP50-95. These experimental results highlight the significant benefit of using transformer-based feature extraction, which contributes to many precise object detections and enhances global context awareness. In summary, the proposed methodology achieves better results than previous studies, ensuring its effectiveness and robustness in handling complex agricultural object detection operations.

**Table 20:** The comparison with related studies on the Taiwan dataset.

Paper	Methodology	Dataset	Classes NO.	Epochs	Precision	Recall	F1-Score	mAP50	mAP50-95
Abulizi et al. ^34^ (2025)	Improved YOLO9	^37^	9	100	92.5	86.6	89.38	95.1	86.4
Bachri et al. ^30^ (2024)	Faster RCNN	^37^	9	100	—	—	—	87.01	—-
Kohut et al. ^28^ (2024)	Faster RCNN (ResNet50)	^37^	9	80	—	—	—	96.5	84
Wang et al. ^29^ (2024)	YOLOv6	^37^	9	100	92.9	95.2	94	93.8	—
Liu et al.^16^ (2025)	Swin	Tomato DS^37^	9	100	83.2	76.5	81.9	84.6	69.3
Dhiab et al.^70^ (2025)	YOLOv8	Subset^37^	9	—	81.9	67.6	74.07	77.4	—
Islam et al.^71^ (2026)	YOLOv9	^37^	9	150	95.8	92.4	94.07	97	93
Proposed methodology	YOLO11n with Deit	^37^	9	100	97.3	93.8	95.5	97.8	93.4

#### Using TLDDCV dataset

The results presented in Table 21 compare the proposed methodology with previous papers applied to the TLDDCV dataset. Although the proposed YOLO11n model with the DeiT transformer backbone demonstrates competitive performance, the improvement margin is relatively limited on this dataset. This limitation is due to the nature of the TLDDCV dataset, which includes low-quality, noisy images and imbalanced class distributions that make precise identification more difficult.

Therefore, various previous papers have depended on this dataset, applying many data augmentation techniques to enhance model generalization and increase image diversity. In contrast, the proposed methodology contained minimal augmentation to evaluate the effectiveness of the modified architecture. In addition to the performance gains on TLDDCV DS not being as significant as on Taiwan DS, the experimental results still evaluate the proposed model’s performance and strengthen the image processing and augmentation techniques for such complex datasets.

The proposed model does really well on the Taiwan dataset. However it does not do well on the TLDDCV dataset. This is because the TLDDCV dataset is harder. The Taiwan dataset and the TLDDCV dataset are different. The TLDDCV dataset has things that make it harder to get good results. First the things in the TLDDCV dataset are more different from each other. They also have backgrounds and the objects are different sizes. Second the Taiwan dataset and the TLDDCV dataset were made in ways. This means the model has a time working well on both datasets. Third the TLDDCV dataset has things that’re harder to find. It is hard to find the objects in the TLDDCV dataset because they are not as clear. This makes it hard to get results when the model has to be very accurate. Finally the people who labeled the objects, in the TLDDCV dataset may not have done it the way. This can also make it harder for the model to do well on the TLDDCV dataset. Even with these problems the model still does well on both datasets. This means the model is pretty good and can work well even when things are different.

**Table 21:** The comparison with the state-of-the-art techniques on TLDDCV dataset.

Paper	Methodology	Dataset	Classes NO.	Epochs	Precision	Recall	F1-Score	mAP50	mAP50-95
Zhang et al. ^27^ (2024)	YOLOv8m & EfficientNetB0	^36^, 737	7	400	0.776	0.751	—	0.790	0.529
Matsui et al. ^31^ (2025)	YOLOv7-tiny	^36^, 737	7	400	—	—	71	70.2	—
AlHusaini et al.^35^ (2025)	YOLOv11	^36^, 737	7		0.80	0.77	—	75.6	—
Proposed Methodology	YOLO11n with Deit	^36^, 737	7	100	83.9	70.3	76.4	73	48

## Limitations and future work

Although the proposed method shows strong performance in the experiments, some limitations should be noted for a balanced view. First, the dataset size is relatively small. This may affect how well the model works with more varied real-world data. Second, class imbalance in some categories might influence the learning process and could lead to bias toward majority classes despite the evaluation strategy used. Third, the computational needs of the proposed framework, including feature extraction and model inference, may grow with larger datasets or real-time deployment. Additionally, while cross-validation has been used to improve generalization, the model has not been tested thoroughly on external datasets. This may limit its ability to generalize across different environments. Future work will focus on addressing these limitations. This will include evaluating the model on larger, more diverse datasets, using better techniques to handle class imbalance, and optimizing the framework’s efficiency for real-time applications and wider deployment.

## Conclusion

This paper presents a comprehensive and novel DL-based system for detecting tomato leaf diseases, solving common challenges in related research, such as low image quality, insufficient feature selection, and unoptimized detection models. This proposed methodology consisted of key components: leaf image processing through the application of a bilateral filter with edge preservation and gamma correction, automatic hyperparameter optimization through a metaheuristic genetic algorithm, and an edited YOLOv11n object detection model with a DeiT transformer backbone for enhanced feature extraction. This system has been evaluated on two public datasets: Taiwan DS (9 classes) and TLDDCV DS (7 classes), achieving better results in precision, recall, F1-score, and mAP metrics. Incorporating a transformer-based backbone enhances the model’s ability to integrate global contextual information with discriminative features, thereby improving detection consistency and accuracy. In addition to applying the genetic algorithm, which enables automatic parameter fine-tuning, it eliminated manual trial-and-error and enhanced efficiency. These outcomes confirm the proposed method’s potential for accurate, real-time detection of tomato leaf diseases in greenhouse, smart agriculture, and field management scenarios. Future work will concentrate on extending the system to additional crop types, enabling multi-class and multi-crop disease detection across many agricultural scenarios. Further developments will focus on low-resource and edge devices, utilizing advanced compression techniques such as quantization and pruning, to minimize computational costs while maintaining accuracy. Further architectural enhancements will also be investigated through modifications to the backbone, Neck, and head components of the YOLOv11n architecture, aiming for improved multi-scale feature aggregation and more precise detection outputs. Optimizing these components could further improve the system’s performance and importance under real-world conditions.

## Data Availability

The datasets used and tested in this study are publicly accessible. The first dataset is the Taiwan dataset, which can be found at the following link: (https://universe.roboflow.com/bryan-b56jm/tomato-leaf-disease-ssoha). The second dataset is the TLDDCV dataset, which can be found at the following link: (https://universe.roboflow.com/sylhet-agricultural-university/tomato-leaf-diseases-detect)

## Code availability

The source code of the proposed framework, including the implementation of the proposed methodology and experimental setup, is publicly available in the Zenodo repository: https://doi.org/10.5281/zenodo.20777853.
